# Mesogenic Groups Control the Emitter Orientation in Multi‐Resonance TADF Emitter Films**

**DOI:** 10.1002/anie.202218911

**Published:** 2023-03-08

**Authors:** Dongyang Chen, Francisco Tenopala‐Carmona, Julius A. Knöller, Andreas Mischok, David Hall, Subeesh Madayanad Suresh, Tomas Matulaitis, Yoann Olivier, Pierre Nacke, Frank Gießelmann, Sabine Laschat, Malte C. Gather, Eli Zysman‐Colman

**Affiliations:** ^1^ Organic Semiconductor Centre EaStCHEM School of Chemistry University of St Andrews St Andrews Fife KY16 9ST UK; ^2^ Humboldt Centre for Nano- and Biophotonics Department of Chemistry University of Cologne Greinstr. 4-6 50939 Köln Germany; ^3^ Institut für Organische Chemie Universität Stuttgart Pfaffenwaldring 55 70569 Stuttgart Germany; ^4^ Laboratory for Chemistry of Novel Materials University of Mons Mons Belgium; ^5^ Laboratory for Computational Modeling of Functional Materials Namur Institute of Structured Matter Université de Namur Rue de Bruxelles 61 5000 Namur Belgium; ^6^ Institut für Physikalische Chemie Universität Stuttgart Pfaffenwaldring 55 70569 Stuttgart Germany

**Keywords:** Liquid Crystals, OLEDs, Thermally Activated Delayed Fluorescence, Transition Dipole Moment

## Abstract

The use of thermally activated delayed fluorescence (TADF) emitters and emitters that show preferential horizontal orientation of their transition dipole moment (TDM) are two emerging strategies to enhance the efficiency of OLEDs. We present the first example of a liquid crystalline multi‐resonance TADF (MR‐TADF) emitter, **DiKTa‐LC**. The compound possesses a nematic liquid crystalline phase between 80 °C and 110 °C. Importantly, the TDM of the spin‐coated film shows preferential horizontal orientation, with an anisotropy factor, *a*, of 0.28, which is preserved in doped poly(vinylcarbazole) films. Green‐emitting (*λ*_EL_=492 nm) solution‐processed OLEDs based on **DiKTa‐LC** showed an EQE_max_ of 13.6 %. We thus demonstrate for the first time how self‐assembly of a liquid crystalline TADF emitter can lead to the so‐far elusive control of the orientation of the transition dipole in solution‐processed films, which will be of relevance for high‐performance solution‐processed OLEDs.

## Graphical abstract

Our work shows how the distinct self‐assembly of a liquid crystalline MR‐TADF emitter can be exploited for generating (partially) aligned via solution‐processed thin films – an advance towards high performance solution‐processable organic semiconductors.

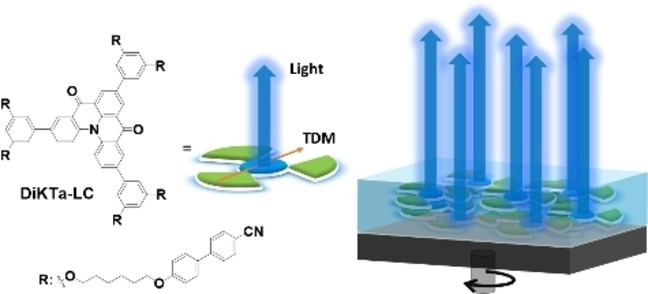

## Introduction

Organic light‐emitting diodes (OLEDs) can be categorized into vacuum‐deposited OLEDs (VD‐OLEDs) and solution‐processed OLEDs (SP‐OLEDs), depending on the technique used for their fabrication.^[1]^ For VD‐OLEDs, with appropriate heating and choice of materials, the vacuum‐deposited films can exhibit high densities, high thermal stability, and the emitter molecules can show a high degree of horizontal molecular orientation.[^1^, ^2^] These properties are critical for the device to be stable and to show a high maximum external quantum efficiency (EQE_max_). However, this fabrication process is energy‐intensive and material‐wasteful, and a complex operation process is required to control doping concentration and film thickness, which contributes to the relatively high fabrication cost associated with VD‐OLEDs.^[3]^ By contrast, SP‐OLEDs offer a number of potential advantages, such as low‐cost manufacturing, high processing efficiency, a relatively small amount of wasted material, and a wider choice of materials.[^3^, ^4^] However, the poor film quality and morphology fabricated by solution‐processed methods lead to inferior device lifetime and severe efficiency roll‐off of SP‐OLEDs, which has, in part, retarded the commercialization of SP‐OLEDs.^[3]^

The efficiency of the OLEDs is based partly on the capacity of the emitter to harvest both the emissive singlet and triplet excitons generated within the emission layer to produce light, which is reflected in the internal quantum efficiency (IQE). There are two classes of materials that can attain up to 100 % IQE: phosphorescent emitters^[5]^ and thermally activated delayed fluorescence (TADF) emitters.^[6]^ Organic TADF emitters harvest all excitons as a result of the small energy gap between singlet and triplet states (Δ*E*_ST_), which permits the non‐emissive triplets to be efficiently up‐converted into emissive singlets by a reverse intersystem crossing (RISC) process.^[7]^ The EQE of the OLED depends not only on the IQE, but also on the light out‐coupling efficiency (*η*_out_), as shown in equation 1:^[8]^

(1) $$EQE=IQE\times \eta_{out}$$

The *η*_out_ is dependent on the orientation of the transition dipole moment (TDM) of the emitters. For the case where the TDMs are randomly oriented within the emissive layer, the *η*_out_ typically has a value of 20–30 %.^[9]^ A general method to improve η_out_ is to horizontally orient the TDM of the emitters, as the emission of light proceeds predominantly in a direction perpendicular to the TDM.^[10]^ There are now a number of examples of VD‐OLEDs that contain highly horizontally oriented TADF emitters.^[2b]^ For SP‐OLEDs, the TDM in polymer films were found to show preferential horizontal orientation even before TDM orientation was investigated in VD‐OLEDs.^[11]^ However, when using small molecule‐based emitters for SP‐OLEDs, the absence of the strong molecular anisotropy imposed by polymer chains and the simultaneous condensation and solidification of the material during the spin‐coating often lead to isotropic TDM orientation in the final film.[^2c^, ^12^] Moreover, the solvent volatilization leaves voids in the film, which provides sufficient space for the emitters to re‐orient to a thermodynamically more stable configuration, often resulting in a net isotropic orientation.^[12]^ Thus, unlike emitters that can show preferential horizontal orientation in vacuum‐deposited films, small molecule emitters in solution‐processed films do not usually show any preferred orientation.^[13]^ A strategy to obtain highly horizontally oriented TDMs in solution‐processed films must exploit intermolecular interactions to drive the assembly of higher order films.

**Figure 1:**
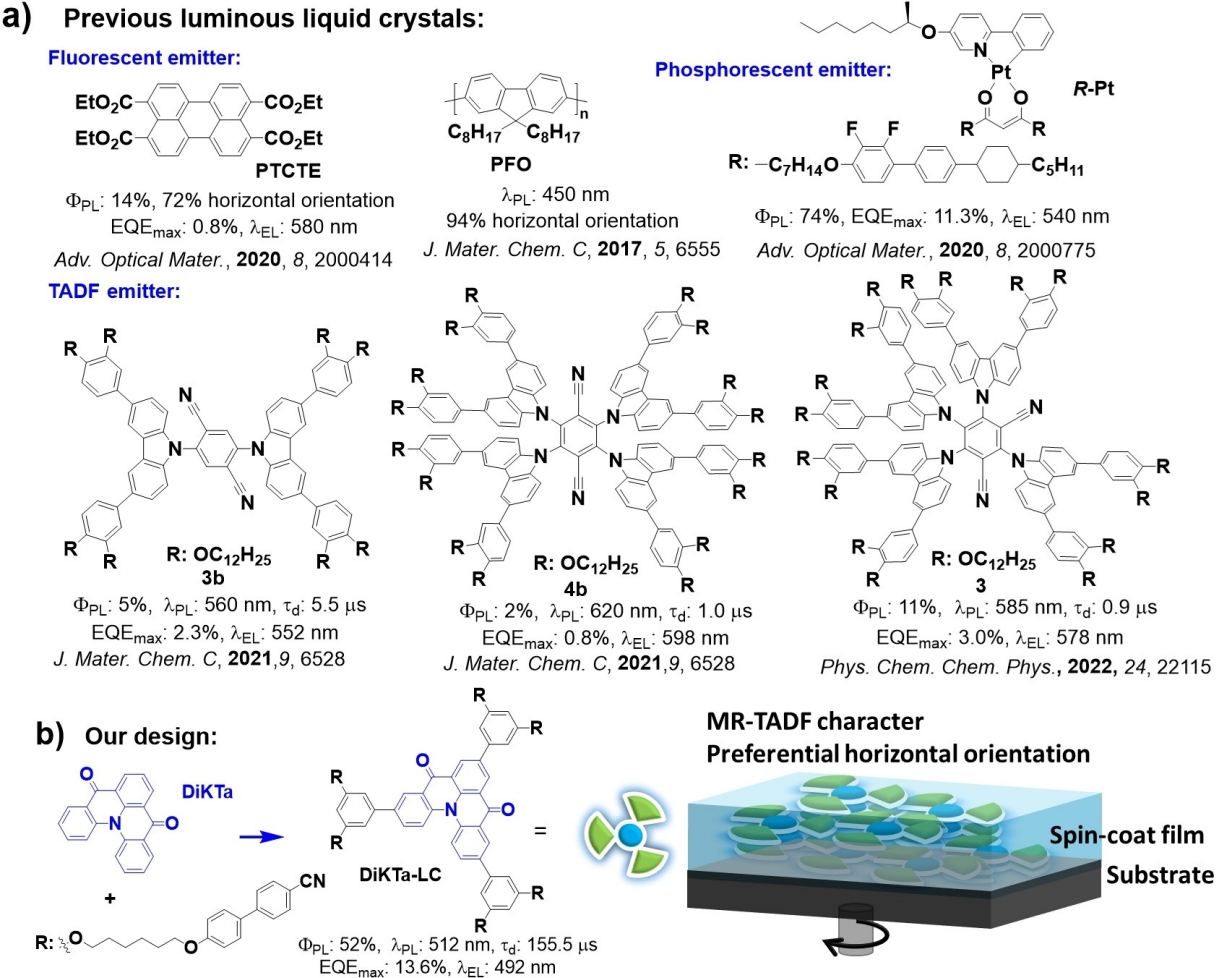
Molecular structures of a) previously reported luminous liquid crystals and b) our MR‐TADF liquid crystal, **DiKTa‐LC**.

Liquid crystalline (LC) materials can form highly ordered microstructures via intermolecular interaction and give rise to anisotropic properties.^[14]^ The alignment direction of liquid crystalline materials can be controlled by simple thermal treatment, electrical field treatment, and mechanical force treatment.[^14^, ^15^] Luminous LC materials, like fluorescent LC emitters^[16]^ and phosphorescent LC emitters^[17]^ have been used in OLEDs due to their controllable photophysical and thermal properties, and charge carrier mobility.[^16b^, ^18^] Although there are examples of the use of both organic fluorescent and metal‐based phosphorescent liquid crystals, to date there are only a few reports of liquid crystalline TADF emitters.^[19]^ Bruce et al. presented the first two examples of TADF emitters **3 b** and **4 b** showing columnar liquid crystalline character by connecting alkoxy chains to the TADF emitting core.^[19a]^ Another TADF liquid crystal, **3**, based on the widely studied TADF emitter **4CzIPN** ^[7]^ was investigated by the same group.^[19b]^ However, the orientation of the TDM of these compounds was not investigated, and their rather low photoluminescence quantum yields (Φ_PL_s) (5 %, 2 %, and 11 % in toluene for **3 b**, **4 b**, and **3**, respectively) contribute to the low efficiency of OLEDs based on them (Figure 1). Recently, Duan et al. showed that by attaching flexible chains with terminal 9,9′‐spirobi[fluorene] (HSF) units to a known TADF emitter core can contribute to a more horizontal orientation of the TDM by increasing the effective planarity of the compound.^[19d]^ The TDM of **5CzBN‐HSF** is 72 % horizontally oriented in the neat film, while the referencing compound, **5CzBN‐Hex** (without HSF units), possesses an isotropically orientated TDM.^[19d]^ These studies opened a new horizon for the use of liquid crystalline materials in OLEDs, especially in terms of the tuning of the film morphology and molecular orientation.

Here, we report the first example of a liquid crystalline multi‐resonant TADF (MR‐TADF) emitter. The structure is based on our previously reported MR‐TADF compound **DiKTa**,^[20]^ which was elaborated with mesogenic groups consisting of 1,6‐dioxyhexyl‐[1,1′‐biphenyl]‐4‐carbonitrile chains resembling the class of mixed rod‐disk mesogens. The compound **DiKTa‐LC** (Figure 1) exhibits MR‐TADF character with delayed lifetime (*τ*_d_) of 70.2 μs, and narrow emission spectra with FWHM=53 nm and *λ*_PL_=514 nm, as a neat thin film with a Φ_PL_ of 41 %. The liquid crystalline character of **DiKTa‐LC** was confirmed by polarized optical microscopy (POM), differential scanning calorimetry (DSC), wide angle and small angle X‐ray scattering (WAXS and SAXS) as the material displays a nematic mesophase between 80 °C and 110 °C. The pristine spin‐coated neat film of **DiKTa‐LC** shows preferential horizontal orientation with an anisotropy factor *a*=0.28 (72 % horizontal), which is preserved after annealing at 100 °C.

## Results and Discussion

**Figure 2:**
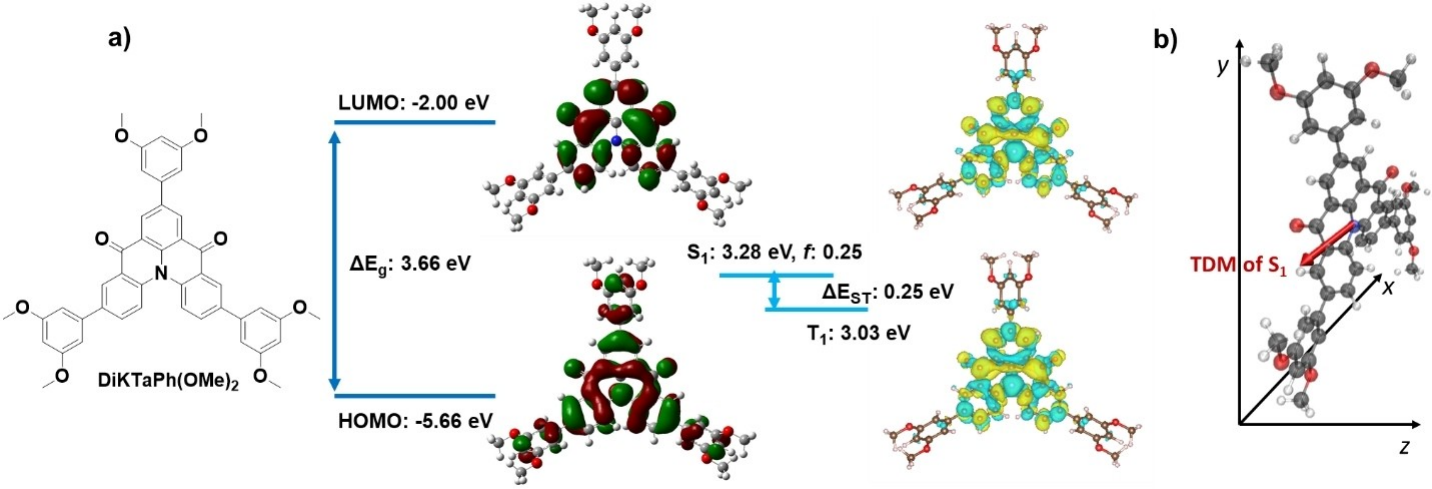
a) Isocontour plots of the HOMO and LUMO orbitals, frontier orbitals energy diagram, difference density plots and energies of the lowest singlet and triplet excited states for **DiKTaPh(OMe)_2_**. b) S_1_ state TDM vector of **DiKTaPh(OMe)_2_**.

We first wished to establish the optoelectronic properties of the MR‐TADF emitter core and how these differ from the reference compound, **DiKTa**. We thus modelled the optoelectronic properties of a model system, **DiKTaPh(OMe)_2_**, by spin‐component scaling second‐order approximate coupled‐cluster (SCS‐CC2) calculations, which we have previously shown to be more accurate than time‐dependent density functional theory.^[21]^ We also modelled the ground state electronic structure at the PBE0/6‐31G(d,p) level of density functional theory (DFT) for both **DiKTaPh(OMe)_2_** and **DiKTa‐LC**.^[22]^ The electron density distribution of the HOMO and LUMO obtained by DFT of **DiKTaPh(OMe)_2_** and **DiKTa‐LC** are similar, while the difference density plots obtained by SCS‐CC2 of **DiKTaPh(OMe)_2_** show the characteristic alternating pattern associated with MR‐TADF compounds. The alkyl‐linked cyanobiphenyl units of **DiKTa‐LC** are not involved in the HOMO and LUMO distributions. The HOMO and LUMO energies of **DiKTaPh(OMe)_2_** are −5.66 eV and −2.00 eV, respectively, while the HOMO energy of **DiKTa‐LC** is 0.33 eV stabilized (−5.99 eV) and LUMO energy is 0.38 eV stabilized (−2.38 eV) (Figure S1). As a result, the HOMO/LUMO energy gap (Δ*E*_g_) of **DiKTa‐LC** is slightly narrowed at 3.61 eV compared to 3.66 eV for **DiKTaPh(OMe)_2_** (Figure 2).

Using SCS‐CC2/cc‐pVDZ the energies of the S_1_ and T_1_ states of **DiKTaPh(OMe)_2_** are calculated to be 3.28 eV and 3.03 eV, respectively. The corresponding Δ*E*_ST_ of **DiKTaPh(OMe)_2_** is 0.25 eV, which is modestly decreased compared to that of **DiKTa** and **Mes_3_DiKTa** (0.27 and 0.26 eV, respectively).^[20]^ Like **DiKTa** and **Mes_3_DiKTa**, the T_1_ and S_1_ states are short‐range charge transfer (SRCT) excited states and the oscillator strength (*f*) from S_1_ is predicted to be high (0.22) for **DiKTaPh(OMe)_2_**, which reflects the significant overlap of the electron density distributions of the HOMO and LUMO of the short‐range charge transfer excited state. The TDM vector of **DiKTaPh(OMe)_2_** of the S_1_→S_0_ transition is oriented with a small angle of 8.2° to the plane of the molecule (X/Y plane in Figure 2b) and is almost aligned with the X‐axis. This result indicates that the likely TDM orientation of **DiKTa‐LC** highly co‐aligns with the plane of the **DiKTa** core.

**Scheme 1:**
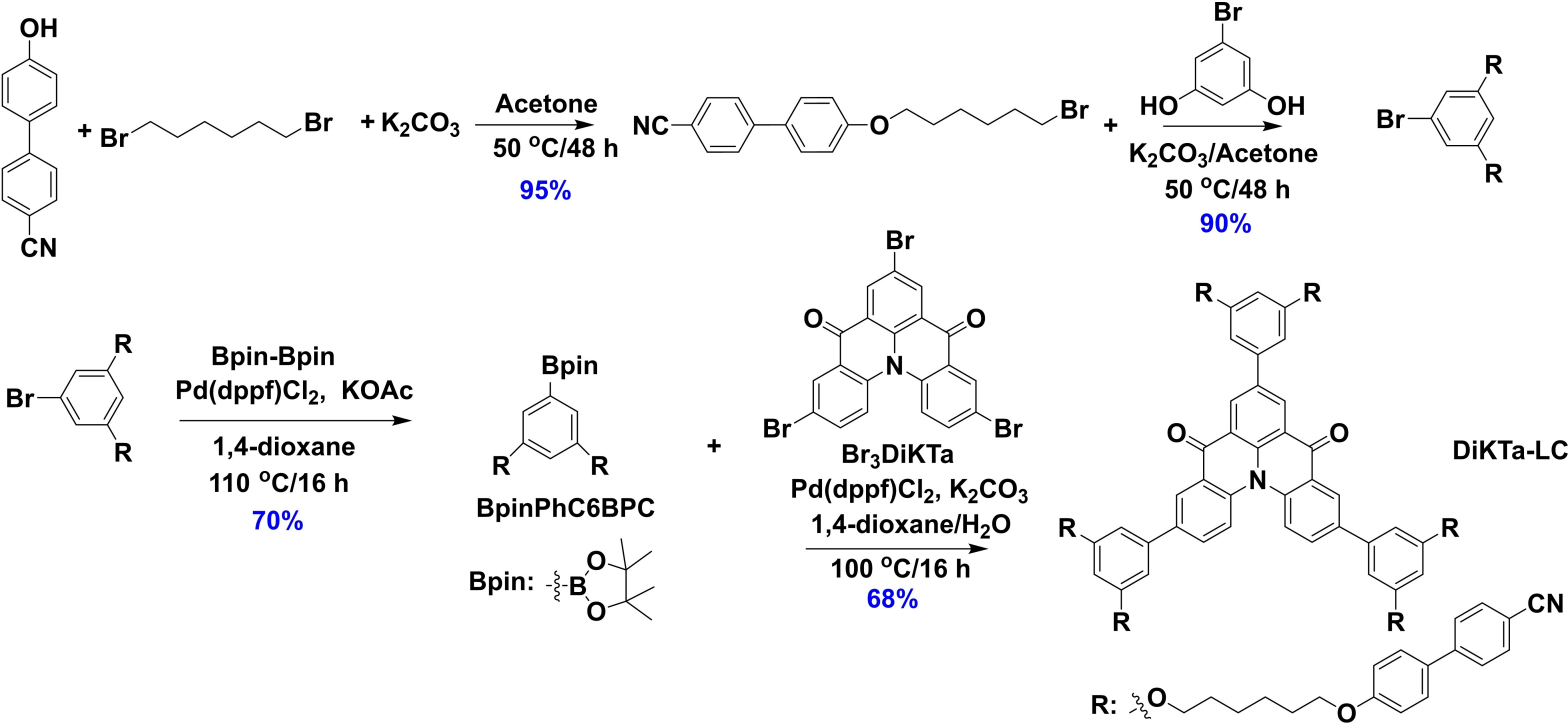
Synthesis of **DiKTa‐LC**.

The intermediate **Br_3_DiKTa** was synthesized following our previously developed protocol,^[20]^ and this was coupled to the mesogenic group, **BPinC6BPC**, under Suzuki–Miyaura cross‐coupling conditions in 68 % yield to afford **DiKTa‐LC**; the mesogenic **BrC6BPC** intermediate was obtained in three steps as outlined in Scheme 1. The identity and purity of the title compound were determined by a combination of ^1^H and ^13^C NMR spectroscopy, high resolution mass spectrometry, melting point, elemental analysis, and high‐performance liquid chromatography.

The electrochemical properties of **DiKTa‐LC** were investigated by cyclic voltammetry (CV) and differential pulse voltammetry (DPV) in DCM using tetrabutylammonium hexafluorophosphate [(*n*‐Bu_4_N)PF_6_] as the supporting electrolyte (Figure S2). The CV trace of **DiKTa‐LC** exhibits irreversible reduction and oxidation waves with *E* ^red^ of −1.49 V and *E* ^ox^ at 1.34 V vs SCE, determined from the DPV. The corresponding HOMO and LUMO values for of **DiKTa‐LC** are −5.68 and −2.84 eV, respectively. Compared to the HOMO/LUMO values of −5.86/−3.26 for **Mes_3_DiKTa**,^[20]^ **DiKTa‐LC** shows more destabilized HOMO and LUMO values, which is ascribed to the electron‐accepting properties of the peripheral groups and also match our DFT calculation.

**Figure 3:**
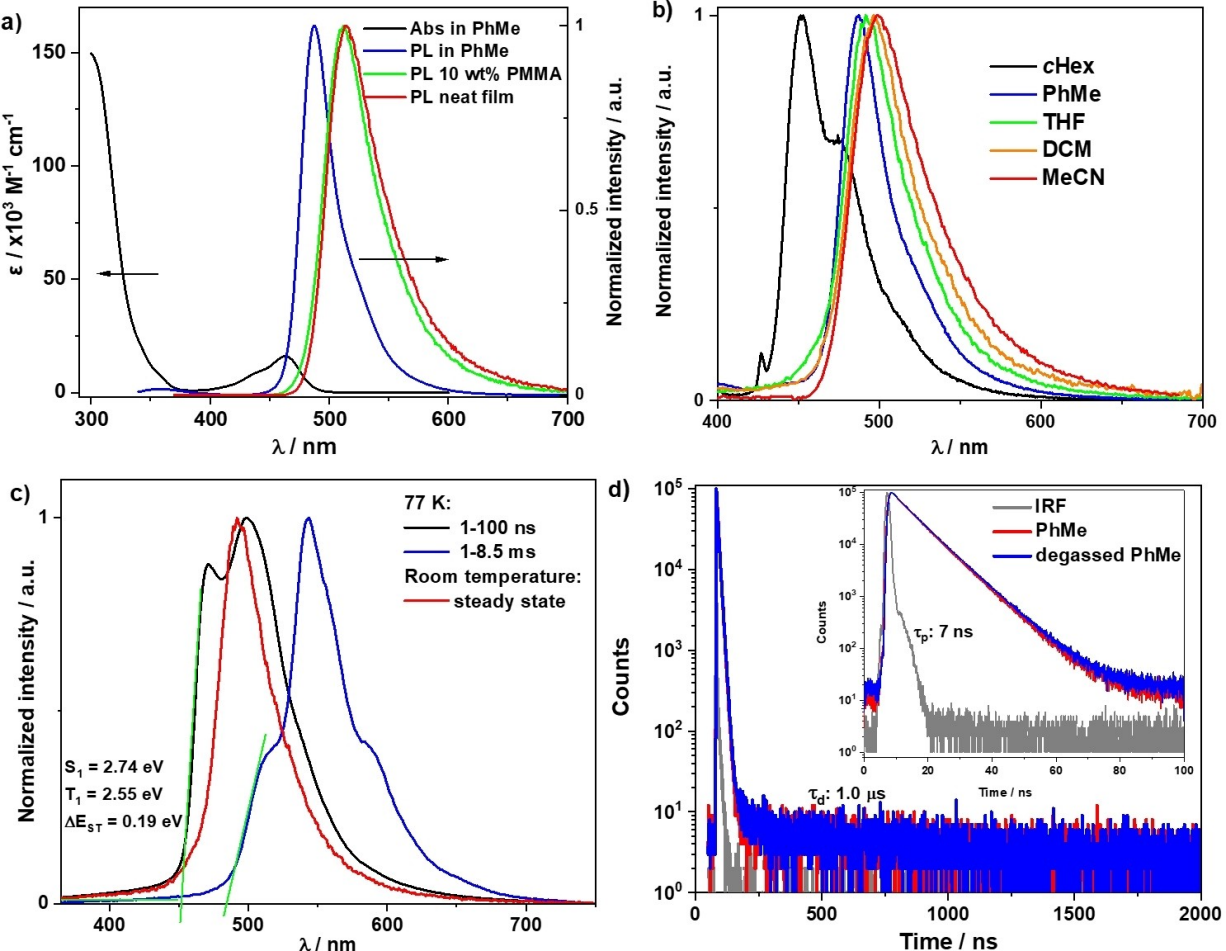
Photophysics of **DiKTa‐LC** in solutions. a) UV/Vis absorption and PL spectra in solution (PhMe) and thin film (10 wt % in PMMA and neat) *λ*_exc_=340 nm. b) Solvatochromic PL shift of **DiKTa‐LC**, *λ*_exc_=340 nm. c) Steady‐state PL at room temperature, prompt PF at 77 K (delay: 1 ns, gate: 100 ns), and phosphorescence spectra at 77 K (delay: 1 ms, gate: 8.5 ms) in 2‐methyl‐tetrahydrofuran, *λ*_exc_=343 nm. d) Time resolved PL of **DiKTa‐LC** in aerate and degassed toluene, *λ*_exc_=379 nm and IRF is the instrument response function of the spectrometer.

The absorption spectrum in toluene of **DiKTa‐LC** (Figure 3a) mirrors those of **DiKTa** and **Mes_3_DiKTa** with a low‐energy band at 464 nm at a molar extinction coefficient (*ϵ*) of around 20×10^3^ M^−1^ cm^−1^ associated with the SRCT state,^[20]^ and a high‐intensity absorption (*ϵ*>100×10^3^ M^−1^ cm^−1^) band at 305 nm attributed to a superposition of locally excited π–π* transitions from the 4‐cyanobiphenyl moieties and the **DiKTa** core.^[23]^ The photoluminescence (PL) spectrum of **DiKTa‐LC** in toluene shows a narrow emission band (FWHM=33 nm) with an emission maximum, *λ*_PL_, of 487 nm, and a small Stokes shift of 24 nm. The Φ_PL_ of **DiKTa‐LC** is 39 % in oxygen‐free toluene, which is comparable to the 37 % measured for **Mes_3_DiKTa**.^[20]^ **DiKTa‐LC** shows structured vibronic progression in nonpolar cyclohexane, whereas in higher polar solvents the emission becomes unstructured and narrowed, except in acetonitrile (MeCN) where it is slightly broadened. The narrowband emission coupled with the small Stokes shift reflect the rigid nature of the emitting core and the small degree of reorganization in the excited state. (Figure 3b). Compared to structureless fluorescence spectra at room temperature in 2‐methyl‐tetrahydrofuran, the prompt fluorescence and phosphorescence spectra at 77 K show a more pronounced structured emission (Figure 3c). The S_1_ (2.74 eV) and T_1_ (2.55 eV) energies of **DiKTa‐LC** were determined from the onset of prompt fluorescence (PF) and phosphorescence spectra. The experimentally determined Δ*E*_ST_ of 0.19 eV is close to the SCS‐CC2 calculated value of 0.25 eV, which is sufficiently small to enable the RISC process. The time‐resolved PL in degassed toluene (Figure 3d) shows a PF lifetime (*τ*_p_) of 7 ns and a *τ*_d_ of 1.0 μs.

**Figure 4:**
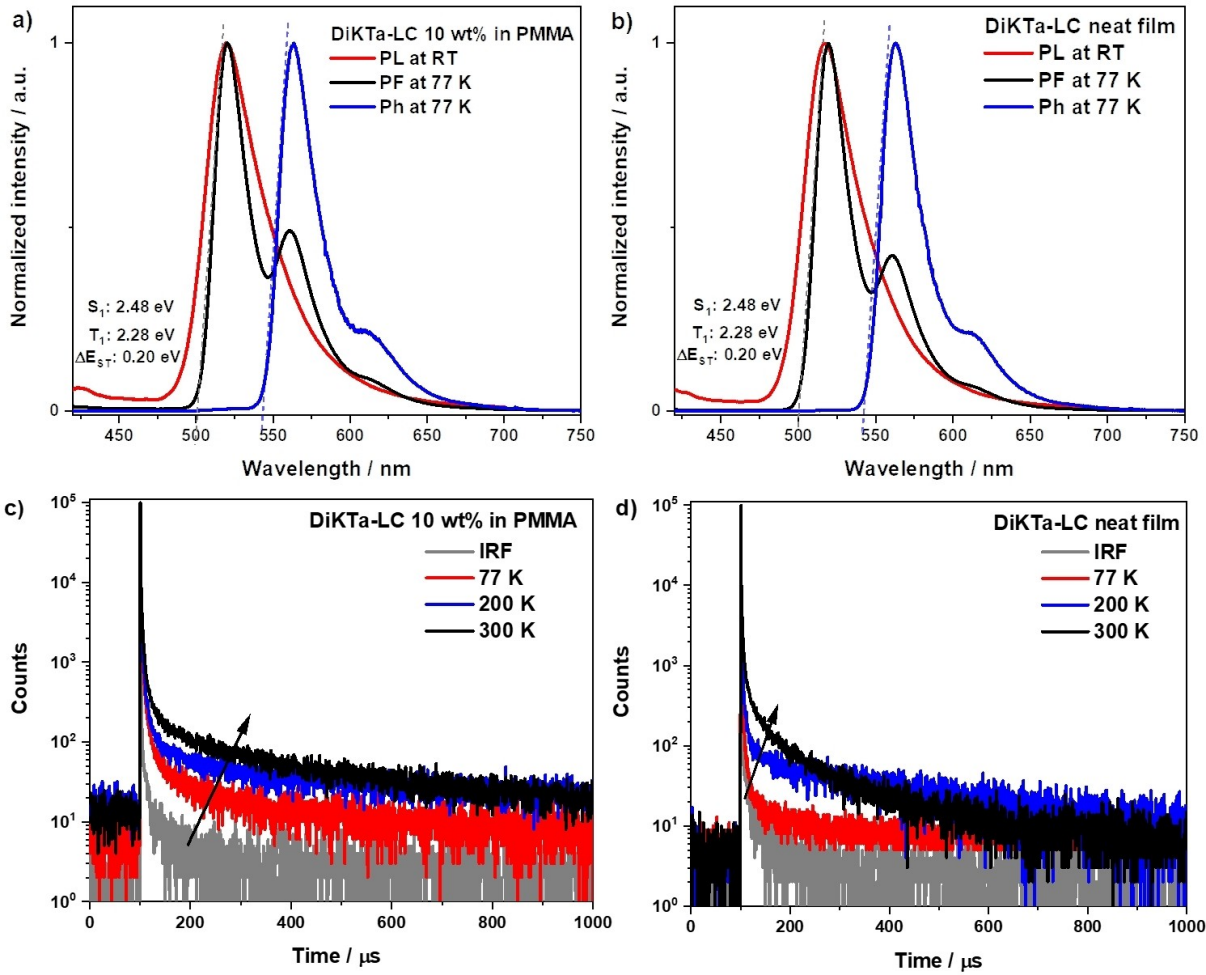
Steady‐state PL spectra at room temperature and prompt fluorescence (PF) at 77 K (delay: 1 ns, gate: 100 ns), and phosphorescence (Ph) spectra at 77 K (delay: 2 ms, gate: 4 ms) of a) 10 wt % doped PMMA film and b) neat film of **DiKTa‐LC**, (*λ*_exc_=390 nm); Temperature‐dependent time‐resolved PL decay spectra of c) 10 wt % doped PMMA film and d) neat film of **DiKTa‐LC** (*λ*_exc_=379 nm).

We next measured the photophysical properties of **DiKTa‐LC** in 10 wt % doped polymethyl methacrylate (PMMA) and neat films. The 10 wt % doped films and neat films of **DiKTa‐LC** show almost identical emission spectra (Figure 4a) that are red‐shifted to 512 nm and 514 nm, and slightly broadened with FWHM of 50 nm and 53 nm, respectively, compared to the PL spectrum in toluene. The Φ_PL_ values of **DiKTa‐LC** in 10 wt % doped PMMA films and neat films are 52 % and 41 %, respectively. The core fragment **DiKTa** features a strong excimer emission in the neat film and strong concentration quenching in doped films. We interpret the absence of an excimer band and strong concentration quenching in **DiKTa‐LC** films due to an efficient separation of the aromatic cores by the mesogenic groups (see below). The 10 wt % doped PMMA films and neat films of **DiKTa‐LC** exhibit almost identical structured steady‐state PL and phosphorescence spectra under 77 K (Figure 4a and b). The S_1_ and T_1_ energies of **DiKTa‐LC** in the solid state (both as doped and neat films), obtained from the onset of the PF and phosphorescence spectra at 77 K, are 2.48 eV and 2.28 eV, respectively, which corresponds to a Δ*E*_ST_ of 0.20 eV. The time‐resolved PL decays of **DiKTa‐LC** (Figures 4c and d) exhibit the same *τ*_p_ of 7 ns in both doped and neat films, while the average *τ*_d_s are 155.5 μs and 70.2 μs, respectively. Temperature‐dependent time‐resolved PL decays reveal that the delayed emission is thermally activated in both doped and neat films.

**Figure 5:**
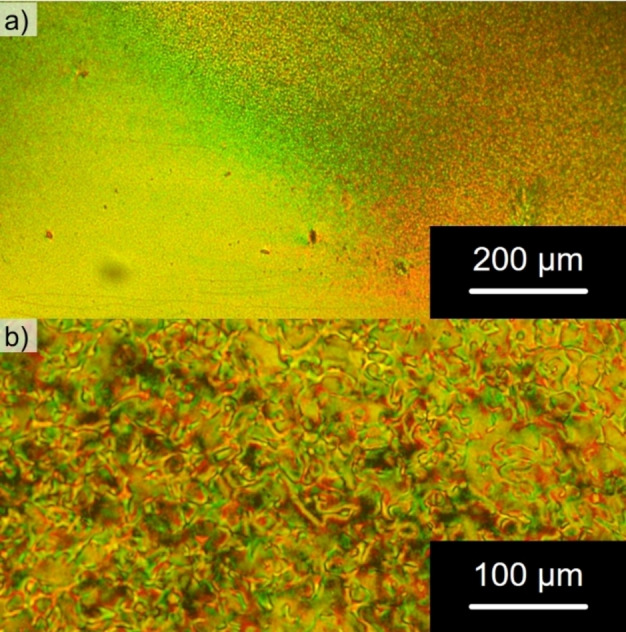
Micrographs of **DiKTa‐LC** obtained on the POM between crossed polarizers upon cooling from the isotropic phase (cooling rate: 1 K min^−1^): a) at a temperature of 90 °C with the sample sandwiched between two glass slides and b) at a temperature of 110 °C with the sample embedded in a cell with a rubbed polyimide coating for heterogeneous alignment.

DSC and POM measurements were conducted to gain insight into the mesomorphic behavior of **DiKTa‐LC** and its precursors. Regarding the precursors, only **BrC6CN** exhibited a monotropic nematic phase (Figures S3–S6).^[24]^ POM investigation of **DiKTa‐LC** revealed mostly uncharacteristic, grainy textures (Figure 5a) that could be easily sheared until they vitrified below 80 °C. Cooling the sample in a cell with a rubbed polyimide coating resulted in thread‐like Schlieren textures (Figure 5b) hinting to the presence of a nematic mesophase.[^24^, ^25^] Other nematic features (two‐ and four‐brush disclinations) were not observed. Upon heating, the DSC trace of **DiKTa‐LC** exhibits a clearing point into the isotropic phase at 110 °C (Figure S6) and then no further transitions. The small clearing enthalpy (1.9 kJ mol^−1^) and entropy (5.0 J K^−1^ mol^−1^) are both within the typical range observed for nematic mesogens of similar structures.[^24^, ^25^, ^26^]

**Figure 6:**
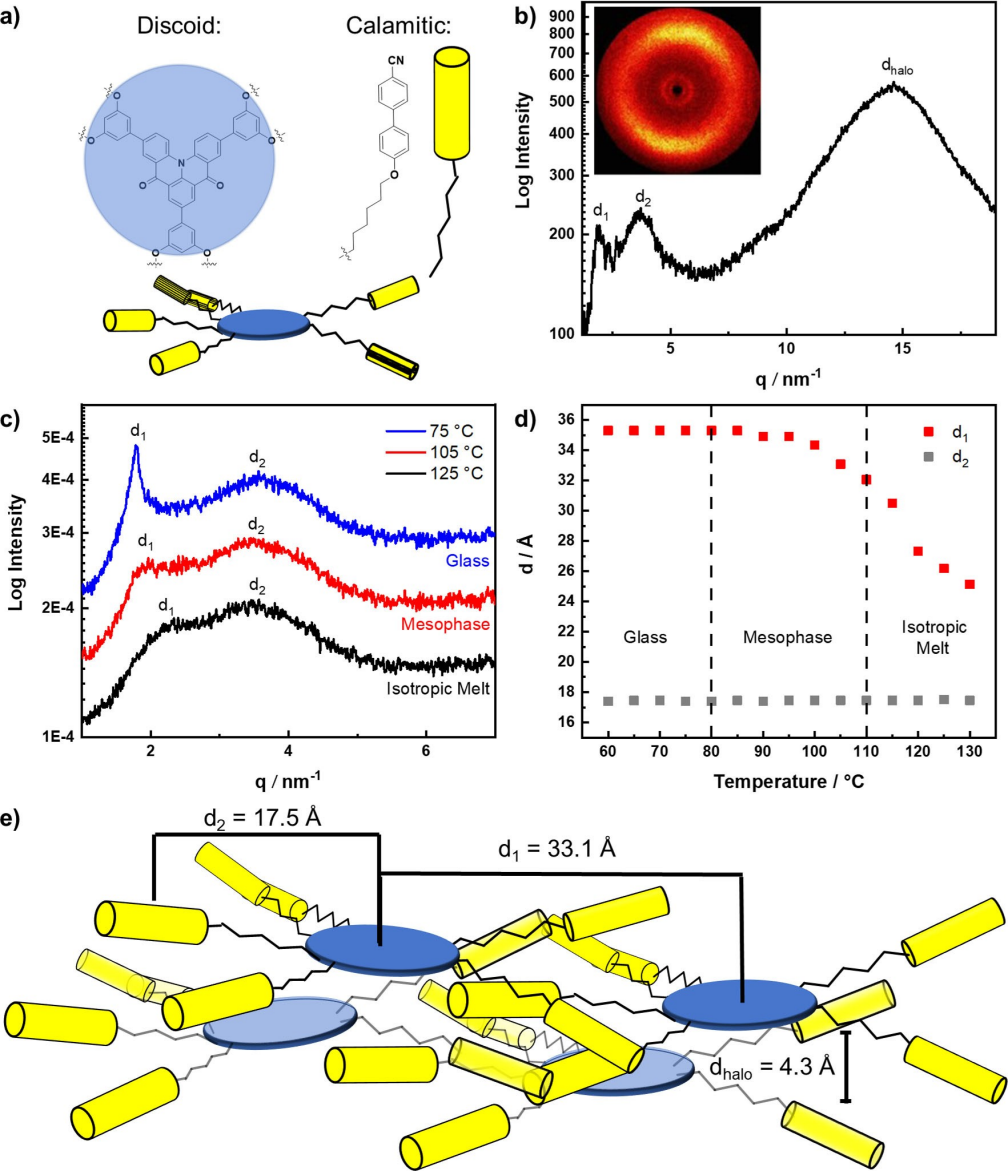
a) Schematic discoid structure of **DiKTa‐LC**, b) WAXS diffractogram of **DiKTa‐LC** recorded at 97 °C upon heating, inset shows the 2D WAXS pattern c) 1D SAXS diffractograms recorded during cooling at 125 °C, 105 °C and 75 °C (*q*_min_=1 nm^−1^, *d*_max_=62.8 Å), d) temperature dependence of *d*_1_ and *d*_2_ deduced from SAXS measurements e) Schematic assembly of **DiKTa‐LC** in a N_D_ phase with the experimental distances *d*_1_, *d*_2_ and *d*_halo_ (not to scale). Please note that the distances *d*_1_, *d*_2_ and *d*_halo_ do not represent spatial correlations, but average distances in the highly disordered nematic phase.

**DiKTa‐LC** is composed of a discotic (**DiKTa**) core decorated with calamitic cyanobiphenyl units and thus resembles the class of shape amphiphilic LCs.^[27]^ Nematic behavior and formation of higher ordered lamellar mesophases by shape amphiphilic mesogens through nanophase segregation into “disk‐” and “rod‐ like” domains has been reported previously, but it requires a non‐symmetric molecular shape for efficient packing.^[26]^ Given the close‐to centrosymmetric and discoid overall shape of **DiKTa‐LC**, a nematic discotic (N_D_) mesophase as observed for similarly substituted triphenylenes seems more likely.^[27d]^ The 2D WAXS pattern of **DiKTa‐LC** at 97 °C (Figure 6b) featured three diffuse peaks and orthogonal alignment of the small‐ and wide‐angle peaks typical for nematic phases.^[26]^ Alignment of the sample occurs spontaneously and we were not able to orient the sample with a magnetic holder. The diffuse halo (*d*_halo_=4.3 Å) corresponds to the short axis distance of the mesogens (Figure 6e) and is quite intense relative to the small angle peaks as observed for similarly substituted triphenylenes.^[27d]^ The first SAXS peak, *d*_1_=33.1 Å (at 105 °C), corresponds to the intermolecular distance (Figure 6e) of the mesogens and was considerably smaller than the calculated molecular diameter of **DiKTa‐LC** (*d*≈51.6 Å, average from DFT‐optimized structure, Figure S10). This indicates strong interdigitation of the alkylcyanobiphenyl units of neighboring **DiKTa‐LC** molecules in the nematic mesophase (Figure 6c) as observed for similarly hexa‐CB‐substituted triphenylenes.^[27d]^ No second SAXS peak was reported for these triphenylenes^[27d]^ but has been documented in other nematic LCs.[^26^, ^28^] Temperature‐dependent 1D SAXS experiments revealed distinct temperature dependence of d_1_ and d_2_ over all three phases (glass, mesophase and isotropic melt, Figure 6c and Figure S8) and thus d_2_ cannot be a higher harmonic of d_1_. Due to the invariance of d_2_ in all three phases, we hypothesize that d_2_ originates from an intramolecular correlation,^[29]^ e.g. the distance between both aromatic **DiKTa** core and the peripheral cyanobiphenyl units (average distance from DFT‐optimized structure: *d*_intra_≈19.3 Å; Figure S10). Additionally, **DiKTa‐LC**, with its large aromatic core, might form columnar stacks in a columnar nematic (Col_N_) mesophase. While the non‐mesogenic chromophore **DiKTa** tends to aggregate in the solid state, resulting in red‐shifted and broadened emission, no such behavior was observed for **DiKTa‐LC** (see above), suggesting relatively weak electronic interactions of the **DiKTa** cores in **DiKTa‐LC**.^[20]^ This result, together with the absence of a π‐π reflection in the XRD data of **DiKTa‐LC** (Figure 6b), prompted us to rule out an aggregated Col_N_ phase in favor of a N_D_ phase with isolated **DiKTa‐LC** molecules, which also takes into consideration the close similarity with Imrie's hexa‐CB‐substituted triphenylene.^[27d]^

The structure of the N_D_ phase is preserved in the glass phase as deduced from similar SAXS and WAXS patterns at 97 °C and 69 °C (Figure S7). These findings prompted us to explore the correlation between the structure of **DiKTa‐LC** and its orientation in solution‐processed films.

The combination of a stable N_D_ phase formed by **DiKTa‐LC** and the close alignment of its main TDM of emission (S_1_→S_0_) with its **DiKTa** core (Figure 2b) can lead to a preferentially horizontal orientation of the latter and, consequently, to an enhanced outcoupling efficiency of **DiKTa‐LC**‐based OLEDs. Therefore, we investigated the influence of the order observed in this N_D_ mesophase on the alignment of its TDM via variable‐angle spectroscopic ellipsometry (VASE) and angle‐resolved photoluminescence spectroscopy (ARPL) measurements of neat films of **DiKTa‐LC** (see section S‐6 for details about sample preparation, measurements, and calculation of the TDM orientation).

The results from the VASE measurements revealed a strong optical anisotropy of the films and a clear preferentially horizontal alignment of the absorption TDM of the transition at ≈290 nm that increased after annealing (Figure S11). We also observed a preferentially horizontal orientation of the absorption TDM of the transition at ≈475 nm [anisotropy factor (*a*)=0.29] that was largely retained after annealing (*a*=0.31). The anisotropy in the orientation of this transition was not as strong as the higher‐energy one. However, it was still clearly observable in these measurements. As the absorption at 290 nm mainly comes from the 4‐cyanobiphenyl moieties of the mesogenic chains, the changes in the anisotropy factor of this transition observed by VASE indicate that there is mesogenic chain reorganization during the annealing process. Such temperature‐dependent reorganization is characteristic of liquid crystals^[30]^ and is consistent with the POM measurements. In turn, the anisotropy factor of the absorption at 475 nm, remained unchanged after annealing. This indicates that annealing of the films led to a more horizontal alignment of the cyano‐biphenyl moieties of the **DiKTa‐LC** molecules, whereas the average alignment of the **DiKTa** cores remained mostly unchanged.

**Figure 7:**
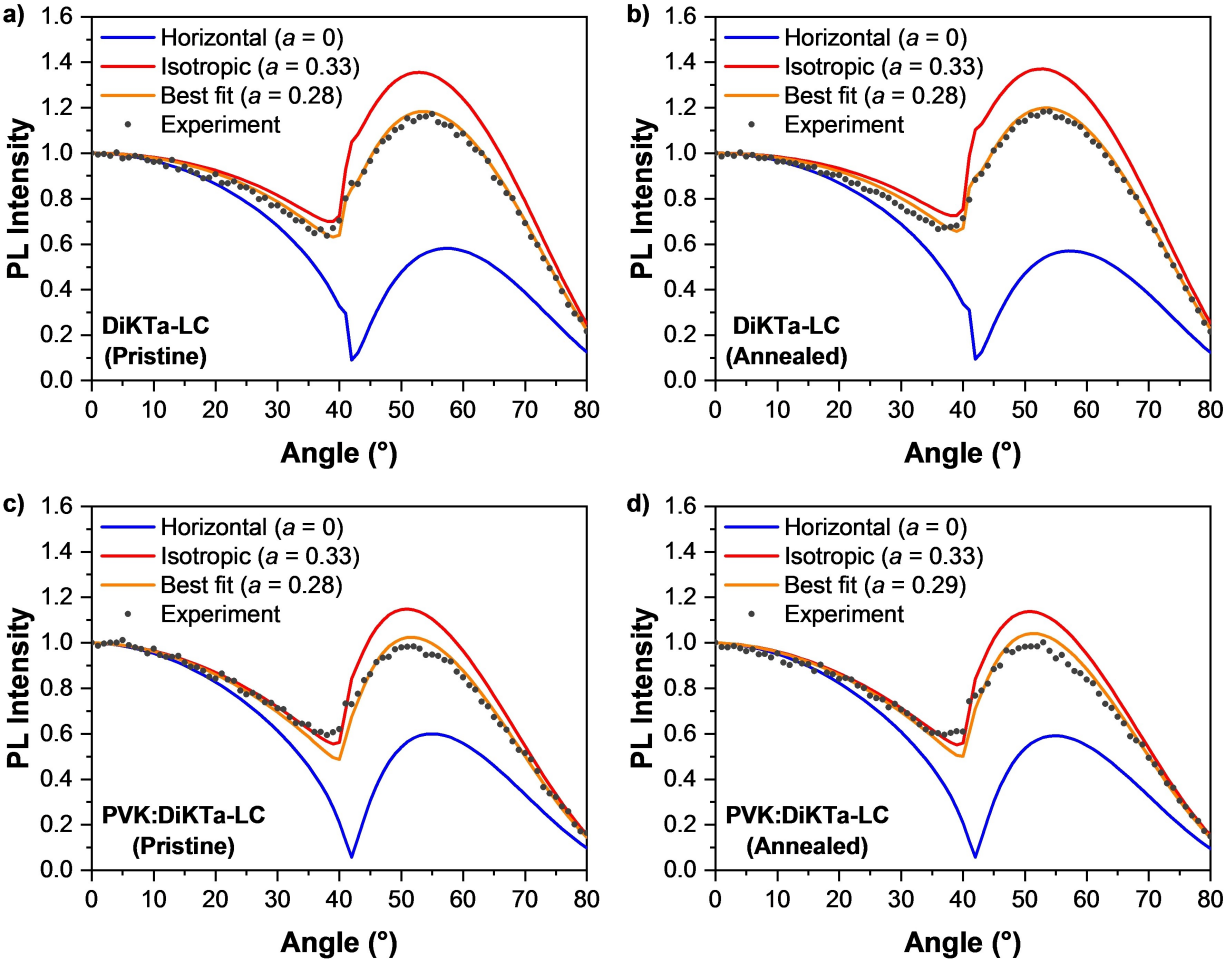
Angular dependence of the PL intensity at the peak emission wavelength of **DiKTa‐LC** films a) pristine and b) annealed. Gray symbols represent the experimental data; lines represent the modelled PL intensity for a perfect horizontal alignment of emitters (blue), isotropic orientation (red), and the best fit to the experimental data (orange). Panels (c) and (d) show the results of the same measurements of pristine (c) and annealed (d) films of films containing 10 wt % **DiKTa‐LC** doped into PVK. All data sets were normalized to the corresponding intensity at 0°.

ARPL spectroscopy measurements probe only the emission from the **DiKTa** core and can thereby provide a more accurate measurement of the orientation of the TDM of emission of the molecules.[^2^, ^31^] The ARPL spectra of the films were measured in a custom‐built setup as described elsewhere.^[32]^ We derived the resulting anisotropy factor *a* by fitting the ARPL at the peak PL wavelength to an optical model based on the transfer‐matrix method.^[33]^ Importantly, we used the anisotropic optical constants obtained from our ellipsometry measurements in this model to reflect the fact that the complex refractive index of the material shows non‐negligible anisotropy.^[33]^ The PL spectra of the annealed films show a slight blue‐shift in the peak wavelength and an increased PL intensity (Figure S12). As shown in Figure 7a, b, both the pristine and annealed films exhibit preferential horizontal orientation with *a*=0.28 (72 % horizontal), which is in good agreement with the result from VASE at 475 nm (Figure S11).

We further verified the impact of the mesogenic groups in **DiKTa‐LC** by comparing the above results to the orientation of spin‐coated neat films of the similar compounds **DiKTa** and **Mes_3_DiKTa** (Table 1). Our VASE results indicated no anisotropy in the optical constants of these films (Figure S13). Unfortunately, the fast photodegradation, low Φ_PL_, and poor morphology of **DiKTa** in neat films made it impossible to obtain reliable results from ARPL. By contrast, anisotropy factors of *a*=0.33 and *a*=0.34 were obtained for pristine and annealed films of **Mes_3_DiKTa**, respectively (Figure S14). These results correspond to an isotropic orientation of the TDMs of emission of this molecule and agree well with the isotropic model obtained from VASE. This implies that the mesogenic groups incorporated into the structure of **DiKTa‐LC** were responsible for the long‐range order and higher degree of horizontal alignment of its TDM.

**Table 1:** Summary of results from ARPL measurements.

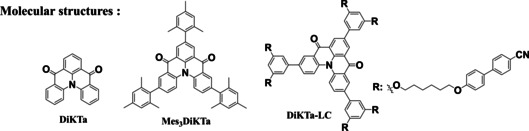			
Emitter	Condition	Anisotropy factor (neat film)	Anisotropy factor (doped film)
**Mes_3_DiKTa**	Pristine	0.33	0.32
	Annealed	0.34	0.33
**DiKTa‐LC**	Pristine	0.28	0.28
	Annealed	0.28	0.29

Finally, we tested whether the TDM orientation of **DiKTa‐LC** can be preserved in doped layers. For this, we spin‐coated films of each emitter (**Mes_3_DiKTa** and **DiKTa‐LC**) doped at 10 wt % in the host polymer poly(n‐vinylcarbazole) (PVK). The results are summarised in Table 1. The anisotropy factors obtained by ARPL indicated isotropic orientations of the TDMs of emission of **Mes_3_DiKTa** (Figure S15). By contrast, anisotropy factors of *a*=0.28 and *a*=0.29 were measured for **DiKTa‐LC** in pristine and annealed doped films, respectively (Figures 7c, d). Remarkably, these values are the same (within the experimental error of our measurements, ±0.01) as those obtained from the ARPL measurements of neat films of **DiKTa‐LC**. While in the neat film this was due to the liquid crystalline properties of this material, it is more likely that the TDM orientation in the doped film is driven also by the anisotropy of PVK (Figure S16), which has birefringent properties.^[34]^ Nonetheless, the anisotropy in the host was not sufficiently strong to induce TDM orientation in **Mes_3_DiKTa**. This implies that the mesogenic groups in **DiKTa‐LC** do aid the alignment of this emitter even in doped solution‐processed films.

**Figure 8:**
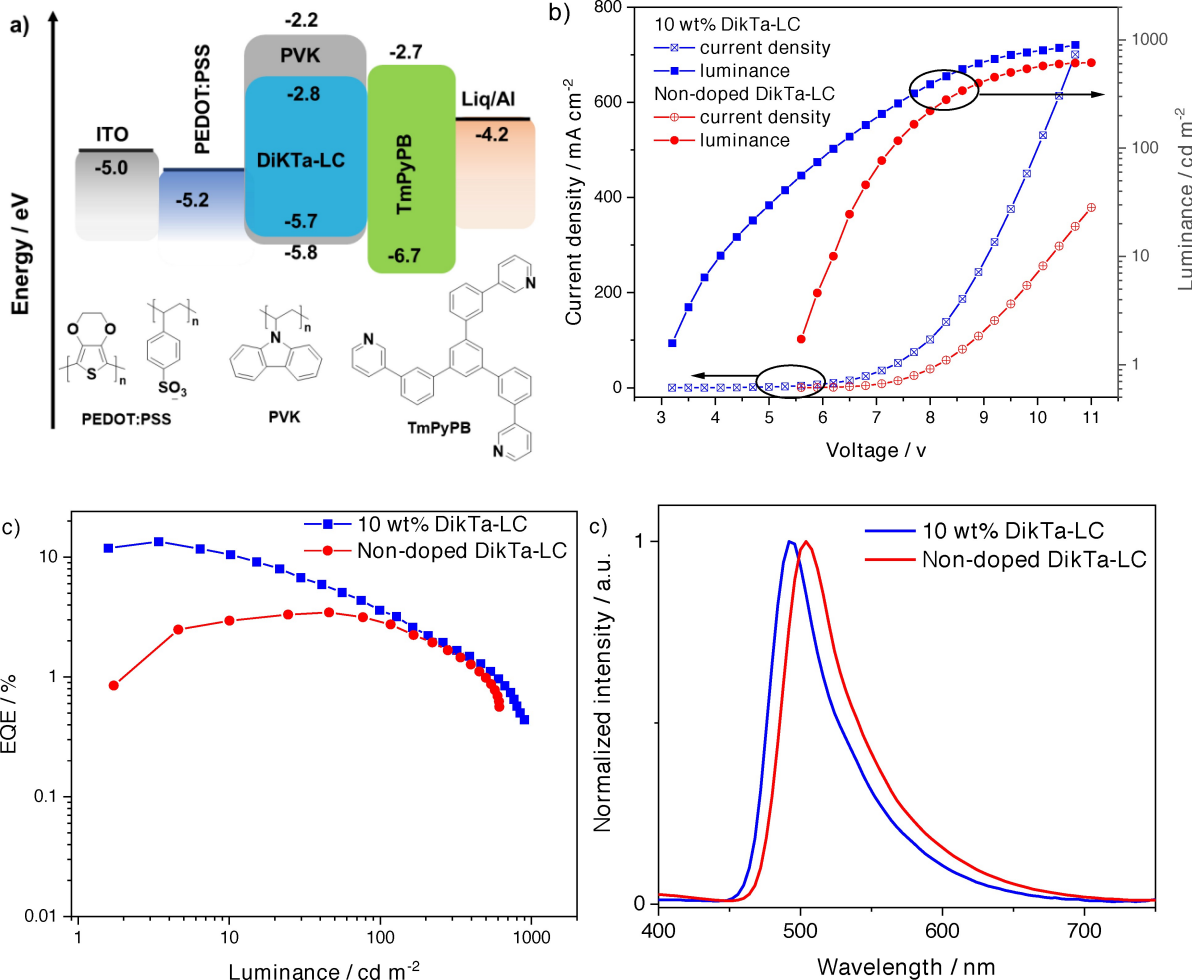
OLEDs based on 10 wt % doped and non‐doped **DiKTa‐LC** emissive layers. a) Proposed energy level diagram of the device structure and molecular structures of the materials used in the device; b) Current density‐voltage‐luminance characteristics; c) EQE‐luminance characteristics, and d) EL spectra.

SP‐OLEDs with **DiKTa‐LC** were fabricated using the architecture: Indium tin oxide (ITO) (120 nm)/poly(3,4‐ethylenedioxythiophene) polystyrene sulfonate (PEDOT:PSS) (35 nm)/10 wt % **DiKTa‐LC**:PVK or **DiKTa‐LC**/1,3,5‐tri(m‐pyridin‐3‐ylphenyl)benzene (TmPyPB) (40 nm)/lithium quinolin‐8‐olate (Liq) (1 nm)/Al (100 nm). PEDOT:PSS and the EML were deposited by spin‐coating while the other layers were deposited by vacuum sublimation. Consistent with the results obtained in our orientation study, 10 wt % **DiKTa‐LC** in PVK was used as the EML, and the device exhibits a low turn‐on voltage (*V*_on_) of 3.2 V and green, narrowband emission with λ_EL_ of 492 nm and a FWHM of 51 nm, corresponding to Commission International de l’Éclairage (CIE) coordinates of (0.22, 0.49). The EQE_max_ of the device reached 13.6 % at 3.4 cd m^−2^ and the maximum luminance (Lum_max_) reached 1000 cd m^−2^ (Figure 8c). Considering the relatively lower Φ_PL_ of **DiKTa‐LC** in PVK (44 %) at 10 wt % doping, and assuming the charge balance (*γ)* and the efficiency of producing radiative excitons (*η*_r_) to both be unity in the device, the *η*_out_ was estimated to be ca. 31 %, given the EQE_max_ of the device. However, as the alkyl chains of the mesogenic groups in the **DiKTa‐LC** are electronically insulating, a much higher *V*_on_ of 5.5 V is required to light up the non‐doped device, and the EQE_max_ decreased to 3.5 % at 45.7 cd m^−2^, this was coupled with an observed bathochromic shift of the EL, with *λ*_EL_=504 nm, a FWHM of 54 nm and CIE of (0.26,0.57).

## Conclusion

By attaching alkyl‐tethered cyanobiphenyl units around a MR‐TADF emitting core, **DiKTa‐LC**, exhibits the photophysical properties of a MR‐TADF emitter such as narrowband green emission (*λ*_PL_=514 nm, FWHM=53 nm), TADF, moderately high photoluminescence quantum yield (*Φ*_PL_ of 41 %) and additionally a nematic mesophase between 80 °C and 110 °C. The liquid crystalline properties of **DiKTa‐LC** were confirmed by POM, DSC, WAXS and SAXS measurements and the material vitrified in a glassy state below 80 °C retaining the disordered features of the nematic mesophase. The pristine spin‐coated neat film of **DiKTa‐LC** and the film of 10 wt % of **DiKTa‐LC** doped into PVK both show preferential horizontal orientation of the TDM and are resistant to thermal treatment at up to 100 °C; this alignment was ascribed to the presence of mesogenic groups in the molecule by direct comparison with the properties of similar compounds **DiKTa** and **Mes_3_DiKTa**. The solution‐processed OLEDs with an EML consisting of a 10 wt % **DiKTa‐LC** PVK film exhibits narrowband green emission, with *λ*_EL_ of 492 nm, a FWHM of 51 nm and CIE of (0.22,0.49). The EQE_max_ reached 13.6 % at 3.6 cd m^−2^, which indicates over 31 % for *η*_out_. Our research shows that the distinct self‐assembly of a liquid crystal can actually be harnessed to control the molecular TDM orientation in solution‐processed films and devices and paves the way towards improved light outcoupling in solution‐processed OLEDs.

## Conflict of interest

The authors declare no conflict of interest.

1

## Supporting information

As a service to our authors and readers, this journal provides supporting information supplied by the authors. Such materials are peer reviewed and may be re‐organized for online delivery, but are not copy‐edited or typeset. Technical support issues arising from supporting information (other than missing files) should be addressed to the authors.

Supporting Information

Supporting Information

## Data Availability

The research data supporting this publication can be accessed at https://doi.org/10.17630/30e801b6‐8f6b‐4779‐8103‐b364e485262d
