# Supplementary material for: Mesogenic Groups Control the Emitter Orientation in Multi‐Resonance TADF Emitter Films
Source: Angew Chem Int Ed Engl. 2023 Mar 8;62(16):e202218911. doi: 10.1002/anie.202218911 (PMC10947294; doi:10.1002/anie.202218911)
Supplement: Supplementary file 2 — Supporting Information [file ANIE-62-0-s001.pdf]

Angewandte Chemie
Eine Zeitschrift der Gesellschaft Deutscher Chemiker

Supporting Information

Mesogenic Groups Control the Emitter Orientation in Multi-Resonance TADF Emitter Films

D. Chen, F. Tenopala-Carmona, J. A. Knöller, A. Mischok, D. Hall, S. Madayanad Suresh, T. Matulaitis, Y. Olivier, P. Nacke, F. Gießelmann, S. Laschat, M. C. Gather*, E. Zysman-Colman**

Table of Contents

Experimental section S-3

General consideration S-3

Quantum chemical calculations S-3

Electrochemistry measurements S-3

Photophysical measurements S-4

Materials and synthesis S-7

Liquid crystal characteristics studies S-14

Materials and Procedures S-14

DSC studies S-15

2D WAXS and SAXS studies S-16

1D SAXS studies S-17

Characteristics spectra S-18

Experimental section

General consideration

All experiments were carried out with commercial solvents from Fisher Scientific Ltd, except where specifically mentioned. Commercially obtained chemicals were used as received. All manipulations were carried out under an inert atmosphere using standard Schlenk line techniques. ^{1}H NMR, and ^{13}C NMR were recorded at room temperature on a Bruker Avance spectrometer at 400 MHz and 100 MHz, respectively. ^{1}H NMR and ^{13}C NMR spectra were referenced to the residual solvent peaks ($CDCl_3$ = 7.26 ppm for ^{1}H NMR and 77.16 ppm for ^{13}C NMR). The following abbreviations have been used for multiplicity assignments: "s" for singlet, "d" for doublet, "t" for triplet, "m" for multiplet and "br" for broad. Elemental analysis was measured by London Metropolitan University. Samples for high resolution mass spectrum (HRMS) were sent to the National Mass Spectrometry Facility in Swansea (EPSRC) for analysis by nano-electrospray on an Orbitrap instrument.

Quantum chemical calculations

The calculations were performed with the Gaussian 09 revision D.018 suite[1] for the Density Functional Theory (DFT) calculations and with the Turbomole/6.5 package[2] for Spin-Component Scaling Coupled-Cluster second-order approximate Coupled-Cluster (SCS-CC2) calculations. Molecular orbitals isocontour plots were obtained based on the gas-phase ground state-optimized structures using the PBE0 functional[3] and the 6-31G (d, p) basis set[4] in the gas phase. Molecular orbitals in ground state were visualized using GaussView 5.0.[5] Excited state calculations were performed using the SCS-CC2 method with the cc-pVDZ basis set.[6] We first optimized the ground state at the SCS-CC2 level and vertical excitations to excited states were calculated based on the SCS-CC2 optimized ground state-optimized structure. Difference density plots were used to track the change in electronic density between the ground and the singlet or triplet excited states and were visualized using the VESTA package.[7]

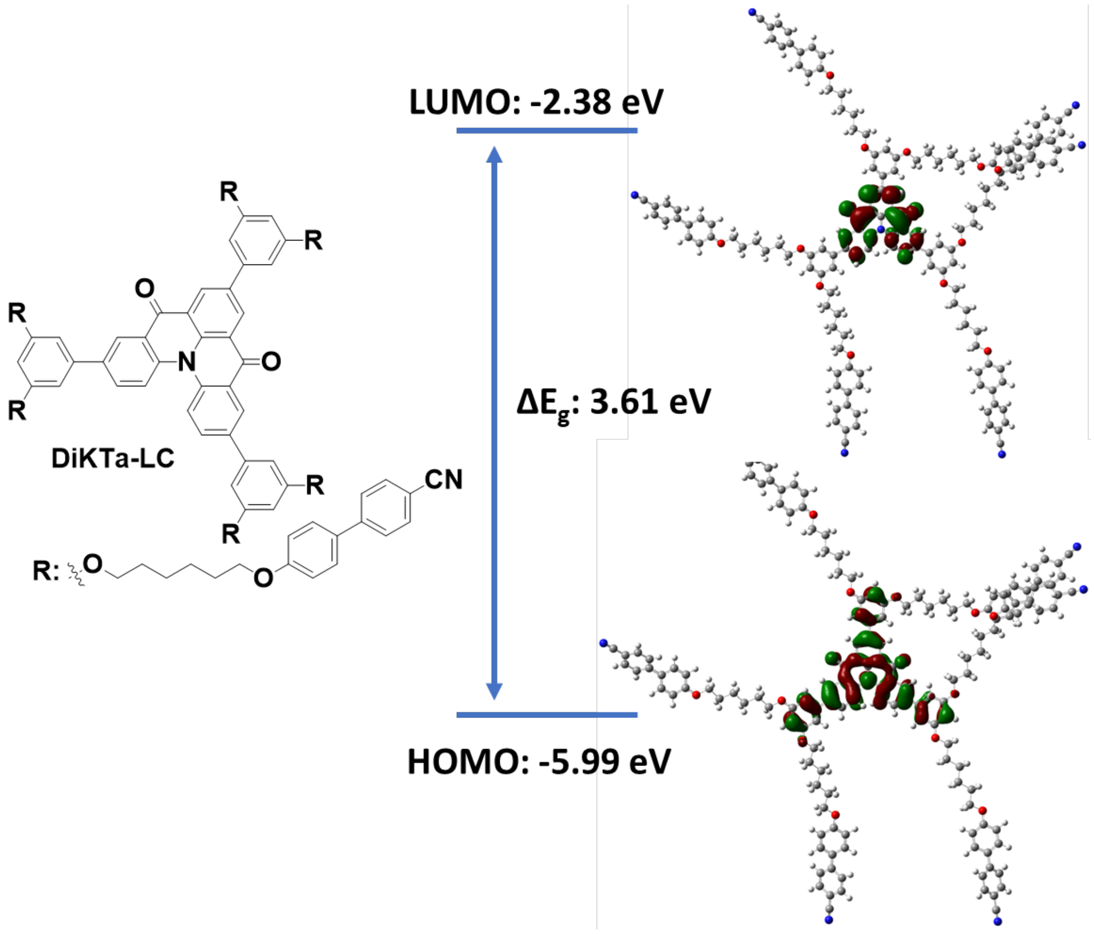

Figure **S1**. Topology of HOMO and LUMO orbitals for **DiKTa-LC**.

Electrochemistry measurements

Cyclic Voltammetry (CV) and Differential pulse voltammetry (DPV) analysis were performed on an Electrochemical Analyzer potentiostat model 620D from CH Instruments. The sample was prepared in dichloromethane (DCM) solution, which was degassed by sparging with DCM-saturated nitrogen gas for 5 minutes prior to measurement. All measurements were performed using 0.1 M tetra-nbutylammonium hexafluorophosphate, $[n\mathrm{Bu_4N}]\mathrm{PF_6}$. An Ag/Ag^+ electrode was used as the reference electrode; a glassy carbon electrode was used as the working electrode and a platinum electrode was used as the counter electrode. The redox potentials are reported relative to a saturated calomel electrode (SCE) with a ferrocene/ferrocenium (Fc/Fc^+) redox couple as the internal standard (0.46 V *vs* SCE).[8] The HOMO and LUMO energies were determined using the relation $E_{HOMO/LUMO} = -(E_{ox} / E_{red} + 4.8)$ eV, where E_{ox} and E_{red} are the values of the anodic and cathodic peak potentials, respectively calculated from DPV relative to Fc/Fc^+.[9]

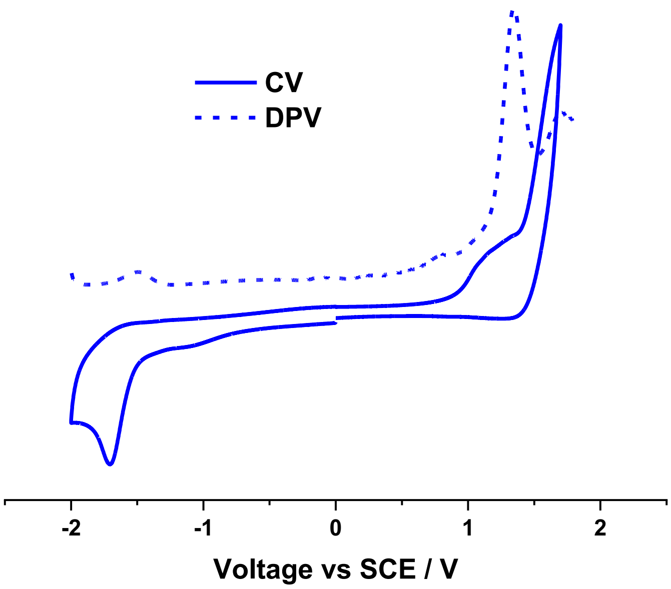

Figure **S2**. CV and DPV traces of **DiKTa-LC** in DCM with 0.1 M *n*-Bu_4NPF_6 as the supporting electrolyte. Measured condition: scan rate 100 mV/s, calibrated against a Fc/Fc^+ redox couple and referenced to 0.46 V versus SCE.[8]

Photophysical measurements

All samples were prepared in HPLC grade solvents with varying concentrations on the order of 10^{-5} or 10^{-6} M for solution absorption and emission study. Absorption spectra were recorded at room temperature using a Shimadzu UV-1800 double beam spectrophotometer with a 1 cm quartz cuvette. Molar absorptivity determination was verified by linear least-squares fit of values obtained from at least five independent solutions at varying concentrations on the order of 1.17×10^{-5} to 5.24×10^{-5} M. For emission studies, aerated solutions were bubbled by compressed air for 5 minutes and degassed solutions were prepared via three freeze-pump-thaw cycles and spectra were taken using home-made Schlenk quartz cuvette. Steady state emission and excitation spectra and time-resolved emission spectra were recorded at 298 K using an Edinburgh Instruments F980 fluorimeter. Samples were excited at 340 nm using a Xenon lamp for steady-state measurements and at 379 nm using a pico-second laser (PicoQuant, LDH-D-C-375) driven by a laser driver (PDL 800-D) for time-resolved emission spectra. The singlet-triplet splitting energy, ΔE_{ST}, was determined from the energy difference between the onsets of the

prompt fluorescence and the delayed phosphorescence spectra in 2-methyltetrahydrofuran glass at 77 K using a Jasco FP-8600 fluorimeter. Photoluminescence quantum yields for solutions were determined using the optically dilute method[10] in which four sample solutions with absorbances of ca. 0.10, 0.075, 0.050 and 0.025 at 340 nm were used. Their emission intensities were compared with those of a reference, quinine sulfate, whose quantum yield (Φ_r) in 1 N H_2SO_4 was determined to be 54.6% using absolute method.[11] The quantum yield of the sample, Φ_{PL}, can be determined by the equation $\Phi_{PL}=\Phi_r(A_r/A_s)(I_s/I_r)(n_s/n_r)^2$, where A stands for the absorbance at the excitation wavelength (λ_{exc}=340 nm), I is the integrated area under the corrected emission curve and n is the refractive index of the solvent with the subscripts "s" and "r" representing sample and reference respectively. Film sample was prepared on sapphire substrates and cooled to 77 K in a cold finger cryostat (Oxford Instruments). The sample was photoexcited using a femtosecond laser emitting at 343 nm (Orpheus-N, model: PN13F1). Emission from the sample was focused onto a spectrograph (Chromex imaging, 250 is spectrograph) and detected with a sensitive gated iCCD camera (Stanford Computer Optics, 4Picos) having sub-nanosecond resolution. Prompt fluorescence spectra were integrated by the iCDD for 10 ns–100 ns after the laser excitation. Phosphorescence spectra were integrated by the iCDD for 1 ms–10 ms after the laser excitation. Thin film Φ_{PL} measurements were performed using an integrating sphere in a Hammamatsu C9920-02 system.[11] Samples were excited at 340 nm by a xenon lamp coupled to a monochromator. The output was then fed into the integrating sphere via a fiber, exciting the sample. The PL was collected with a multimode fiber and detected with a back-thinned CCD (BT-CCD) detector. The thin film Φ_{PL}s were then measured by purging the integrating sphere with N_2 gas flow.

Orientation measurements

All films used for orientation measurements were prepared by spin-coating onto substrates cleaned using ultrasonic bath in milli-Q water followed by acetone and isopropanol, and subsequently treated using either UV-ozone or oxygen plasma treatments. Identical films were either measured as spin coated or after annealing at 100 °C for 1 h. Both spin-coating and annealing were carried out inside a nitrogen-filled glove box.

The neat thin films for variable-angle spectroscopic ellipsometry (VASE) measurements were prepared by spin-coating chloroform dilutions of **DiKTa-LC** at different concentrations (10 mg/mL or 20 mg/mL) onto glass (Eagle XG) or silicon substrates at spin speeds of 4000 RPM for 60 s. Under these conditions, the two different concentrations yielded films with thicknesses of ~80 nm and ~160 nm, respectively, as obtained from ellipsometry measurements. Neat films of **DiKTa** and **Mes$_3$DiKTa** for ellipsometry measurements were fabricated by spin-coating from chloroform dilutions (5 mg/mL, 10 mg/mL, and 15 mg/mL) using the same conditions. These concentrations yielded films with thicknesses of ~25 nm, ~50 nm, and ~80 nm for **DiKTa**, and ~40 nm, ~80 nm, and ~130 nm for **Mes$_3$DiKTa**, respectively. Doped films for ellipsometry measurements were fabricated by diluting **DiKTa-LC** or **Mes$_3$DiKTa** in chlorobenzene together with poly(*N*-vinylcarbazole) (PVK) at a weight ratio of 1:9 emitter:PVK and at final concentrations of 10 mg/mL and 20 mg/mL. These concentrations and spin-coating speeds of 3000 RPM for 60 s yielded thicknesses of ~30 nm and ~90 nm for PVK:**DiKTa-LC** films, and of ~35 nm and ~92 nm for PVK:**Mes$_3$DiKTa**, respectively.

The refractive index, extinction coefficient, and thickness of the films were determined from VASE (M2000, J.A. Woollam) and subsequent modeling (via CompleteEase software, J.A. Woollam). Modeling was performed using transmission and VASE measurement on both glass and Si substrates to yield consistent fits to ellipsometric polarization and transmission data. The orientation of the TDM of absorption in the films can be calculated from the local maxima of the extinction coefficients in the horizontal and vertical directions (k_o and k_e) and expressed in terms of the order parameter (S) and anisotropy factor (a),[15,16]

$$S = \frac{k_e - k_o}{k_e + 2k_o} = \frac{3a-1}{2}. \quad (1)$$

a can be interpreted as the fraction of vertical TDMs in the film. VASE and ARPL can quantify the orientation of the average absorption and emission TDM of the films, respectively.[2,3] The results are expressed in terms of the anisotropy factor (a), which is defined as:

$$a = \langle cos^2\theta \rangle. \quad (2)$$

where θ is the angle between the TDM and the normal to the film, and the brackets denote averaging over the ensemble of individual molecules contributing to the absorption or emission process. Therefore, a can be interpreted as the fraction of vertical TDMs in the film; a perfectly isotropic (randomly oriented) film would yield a value of $a = 1/3$, whereas the limit values $a = 0$ and $a = 1$ would correspond to perfectly horizontal and perfectly vertical orientations, respectively, and $a < 1/3$ indicates a preferentially horizontal orientation. We estimate the error in a to be ± 0.01 (in units of a) for ARPL and ± 0.03 for VASE based on the error in the fits of ARPL and VASE data.

Thin films for angle-resolved photoluminescence (ARPL) measurements were spin-coated onto glass (Eagle XG) substrates using the same conditions described above, but at concentrations of 6 mg/mL and 7 mg/mL for neat films of **Mes_3DiKTa** and **DiKTa-LC**, respectively, and at concentrations of 10 mg/mL for the doped films of both the emitters in PVK. All films used in angle-resolved photoluminescence (ARPL) measurements were encapsulated under nitrogen atmosphere before being taken out of the glove box for measurements.

ARPL spectra of these samples were measured in a custom-built setup as described elsewhere.[12] Excitation was produced by a high-power LED with peak emission at 450 nm (Thorlabs M450LP2) passed through a short-pass filter with cut-off wavelength of 460 nm (Semrock 468/SP BrightLine). The emission from the film was passed through a long-pass filter with a cut-off wavelength of 483 nm (Semrock 473/LP Edge Basic) and collected with an optical fibre coupled to a low-noise spectrometer (OceanInsight QEPro). Although the samples were encapsulated under nitrogen atmosphere, there was noticeable degradation of the films that led to a decrease of the PL intensity during the measurements. Thus, the intensity of the ARPL spectra were adjusted at each angle according to a single-exponential function that was calculated from the difference in the PL spectra at 0° before and after the measurements. This correction avoided an underestimation of the value of the anisotropy factor a, which would have led to an overestimation of the degree of horizontal alignment of the molecules. The resulting value of a was derived by fitting the degradation-corrected angle-dependent PL intensity of the samples at the peak wavelength to an optical model based on the transfer-matrix method.[13] The optical constants obtained from the VASE measurements were used as constant parameters for the

model, while the thickness and anisotropy factor were left as free-fitting parameters. In all cases, the fitted thickness (see Table **S2**) agreed well with the thicknesses obtained from VASE measurements stated above.

Materials and synthesis

Compounds **DiKTa** and **Mes$_3$DiKTa** were provided by Callum Prentice, which were synthesized and purified according to the literature.[14]

N,N-bis(2-methoxycarbonylphenyl)aniline (**MeCA**)

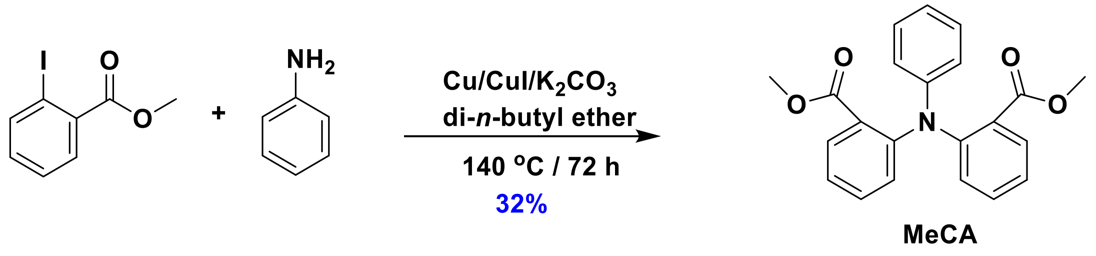

The synthesis of **MeCA** followed our previously reported route.[14] To a 2-neck flask held under nitrogen was charged with aniline (2.3 mL, 25 mmol, 1 equiv.), methyl 2-iodobenzoate (11.0 mL, 75 mmol, 3 equiv.), copper (0.47 g, 7.4 mmol, 0.3 equiv.), copper(I) iodide (0.32 g, 1.73 mmol. 0.07 equiv.), anhydrous potassium carbonate (10.5 g, 75 mmol, 3 equiv.), and 25 mL anhydrous di-*n*-butyl ether. The resulting reaction mixture was heated to reflux and stirred for 3 days. After cooling to room temperature, the reaction mixture was filtered through a pad of celite. The filtrate was then mixed with dichloromethane and washed with water (3 × 50 mL). The organic layer was then dried using anhydrous sodium sulfate and concentrated under reduced pressure. The resulting crude product was purified by column chromatography on silica gel (EtOAc : hexane = 20 : 80). The corresponding fractions were collected and evaporated under reduced pressure to afford the desired product as a white S8 crystalline solid, which was then filtered and washed with hexane.

Yield: 32% (2.9 g). **R$_f$**: 0.54 (EtOAc : hexane = 20 : 80 on silica gel). Mp: 145 °C. **^{1}H NMR (400 MHz, CDCl$_3$) δ (ppm)**: 7.65 (dd, *J* = 7.7, 1.6 Hz, 2H), 7.43-7.39 (m, 2 H), 7.20-7.11 (m, 6 H), 6.88-6.84 (m, 1 H), 6.77-6.75 (m, 2 H), 3.39 (s, 6 H). **^{13}C NMR (100 MHz, CDCl$_3$) δ (ppm)**: 168.0, 148.6, 146.6,

132.8, 131.1, 129.1, 128.8, 128.0, 124.1, 121.8, 120.8, 51.9. **HRMS (ESI-MS) [M+H]$^+$ Calculated**: ($C_{22}H_{19}NO_4$) 362.1387; **Found**: 362.1387. The characterization matches that previously reported.[14]

Dimethyl 6,6'-((4-bromophenyl)azanediyl)bis(3-bromobenzoate) (**3Br-MeCA**)

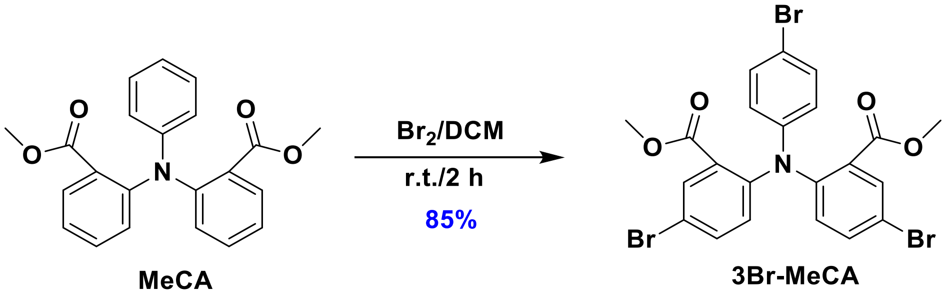

The synthesis of **3Br-MeCA** followed our previously reported route.[14] To **MeCA** (4 g, 11.10 mmol, 1 equiv.) in 100 mL dichloromethane, bromine (1.7 mL, 33.3 mmol, 3 equiv.) was added dropwise. After 1 h stirring at room temperature, further bromine (0.1 mL) was added dropwise, and stirring continued for 0.5 h. The reaction was quenched by the addition of a 10% solution of sodium hydroxide (50 mL). The organic layer was separated, washed with water (3 × 50 mL), dried with anhydrous sodium sulfate and concentrated under reduced pressure. The crude product was purified by flash chromatography on silica gel (10 : 90 EtOAc : hexanes) to afford the **3Br-MeCA** as a light yellow solid.

Yield: 85% (5.6 g). **R$_f$**: 0.45 (10 : 90 EtOAc : hexanes on silica gel). **Mp**: 134 - 136 °C. **^{1}H NMR (500 MHz, $CDCl_3$) δ (ppm)**: 7.81 (d, J = 2.4 Hz, 2H), 7.53 (dd, J = 8.6, 2.4 Hz, 2H), 7.26-7.22 (m, 2H), 7.05 (dd, J = 8.6, 2.4 Hz, 2H), 6.61-6.58 (m, 2H), 3.46 (s, 6 H). **^{13}C NMR (125 MHz, $CDCl_3$) δ (ppm)**: 166.0, 147.1, 144.7, 136.1, 134.1, 132.2, 130.6, 129.4, 122.1, 117.8, 114.8, 52.4. **HRMS (ESI-MS) [M+H]$^+$ Calculated**: ($C_{22}H_{16}{}^{79}Br_3NO_4$); 595.8702; **Found**: 595.8700. The characterization matches that previously reported.[14]

3,7,11-tribromoquinolino[3,2,1-de]acridine-5,9-dione (Br_3DiKTa)

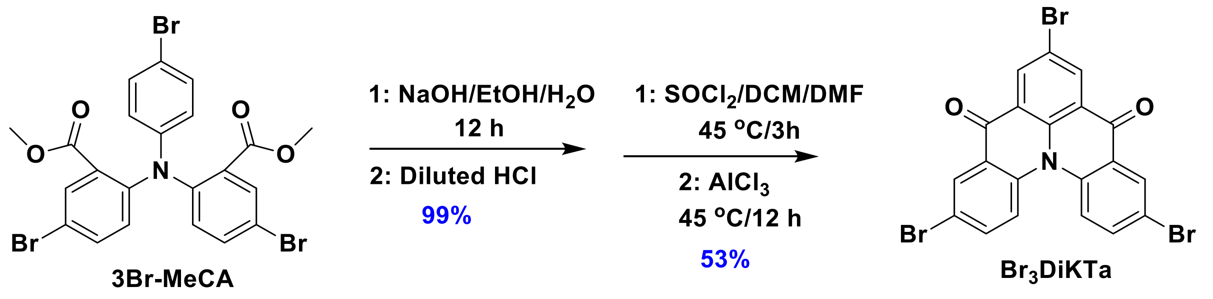

The synthesis of **Br3DiKTa** followed our previously reported route.[14] Compound **3Br-MeCA** (5 g, 8.4 mmol, 1 equiv.) was combined with sodium hydroxide (1.68 g, 42 mmol, 5 equiv.) in 40 mL of an ethanol/water (1/1) mixture. The reaction was heated to reflux for 12 h. After cooling to room temperature, the pH was adjusted to 2-3 by addition of dilute hydrochloric acid. The diacid precipitated as a light green solid and was collected by vacuum filtration, washed thoroughly with water, dried under vacuum (4.75, 99% yield) and used without further purification and characterization. The diacid (4 g, 7.1 mmol, 1 equiv.) was dispersed in 60 mL dichloromethane under a nitrogen atmosphere. To the reaction mixture were added sequentially thionyl chloride (1.03 mL, 14.2 mmol, 2 equiv.) and 7 drops of DMF. After 3 h under reflux, the reaction mixture was cooled to room temperature. Under a positive flow of nitrogen, aluminum chloride (9.46 g, 71 mmol, 10 equiv.) was added slowly (exothermic reaction). After refluxing for 12 h, the reaction mixture was cooled to room temperature and the reaction quenched by dropwise addition of water with vigorous stirring (exothermic reaction). The resulting mixture was combined with dichloromethane (150 mL), the organic layer was then separated. The remaining aqueous layer was washed with dichloromethane (3 × 50 mL) to completely remove the yellow insoluble product from water. The organic fractions were combined, and the solvent volume was concentrated under reduced pressure. The product was filtered and washed with methanol and hexane to afford the desired product as a yellow solid.

Yield: 53% (2.0 g). **M$_p$**: Decomposed at 364 °C. **^{1}H NMR (500 MHz, CDCl3) δ (ppm)**: 8.81 (s, 2H), 8.59 (d, *J* = 2.2 Hz, 2H), 7.95 (d, *J* = 8.9 Hz, 2H), 7.81 (dd, *J* = 8.9, 2.5 Hz, 2H). ^{13}C NMR was not recorded due to poor solubility of this compound. **HRMS (ESI-MS) [M]$^+$ Calculated**: 530.8100 ($C_{20}H_8{}^{79}Br_3NO_2$); Found: 530.8088. The characterization matches that previously reported.[14]

4'-((6-bromohexyl)oxy)-[1,1'-biphenyl]-4-carbonitrile (BrC6CN)

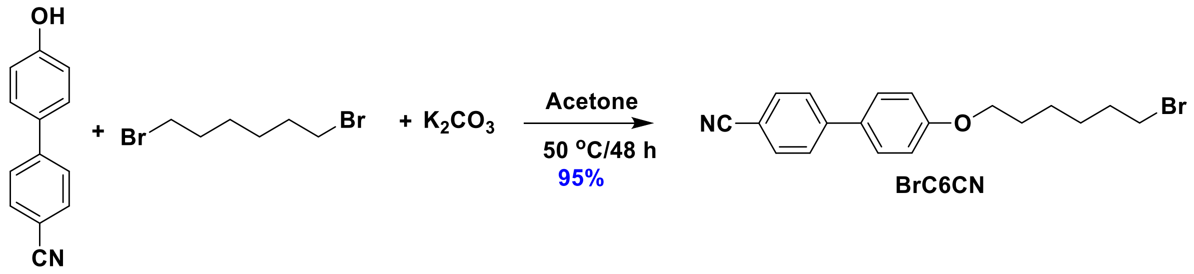

To a 250 mL flask were added 4'-hydroxy-[1,1'-biphenyl]-4-carbonitrile (2.0 g, 10 mmol, 1 equiv.), 1,6-dibromohexane (7.3 g 30 mmol, 3 equiv.), potassium carbonate (4.2, 30 mmol, 3 equiv.). The mixture was degassed by three cycles of vacuum-nitrogen purging and 50 mL of acetone was injected. The mixture was stirred at 50 °C for 24 h under nitrogen atmosphere. After completion of reaction, 50 mL of water was added to the reaction mixture and extracted with DCM (3 × 100 mL). The combined organic layers were dried with anhydrous magnesium sulfate. The organic solvent was removed under reduced pressure and the crude product was purified by silica gel column chromatography. DCM/Hexane=1/4 was used as eluent to afford **BrC6CN** as a white solid.

Yield: 95% (3.4 g). **R_f**: 0.45 (25% DCM/hexanes on silica gel). **Mp**: 56 °C. **^{1}H NMR (400 MHz, $CDCl_3$) δ (ppm)**: 7.75 – 7.62 (m, 4H), 7.55 (d, J = 8.8 Hz, 2H), 7.01 (d, J = 8.8 Hz, 2H), 4.04 (t, J = 6.4 Hz, 2H), 3.46 (t, J = 6.8 Hz, 2H), 2.03 – 1.79 (m, 4H), 1.69 – 1.38 (m, 4H).**^{13}C NMR (101 MHz, $CDCl_3$) δ (ppm)**: 160.8, 159.7, 145.3, 132.6, 131.4, 122.8, 115.1, 110.2, 100.6, 68.3, 31.6, 29.2, 25.8, 22.6, 14.0. **HRMS (ESI-MS) [M+1]$^+$ Calculated**: ($C_{19}H_{20}{}^{79}BrNO$); 358.0807; **Found**: 358.0804.

4',4'''-((((5-bromo-1,3-phenylene)bis(oxy))bis(hexane-6,1-diyl))bis(oxy))bis(([1,1'-biphenyl]-4-carbonitrile)) (BrPhC6BPC)

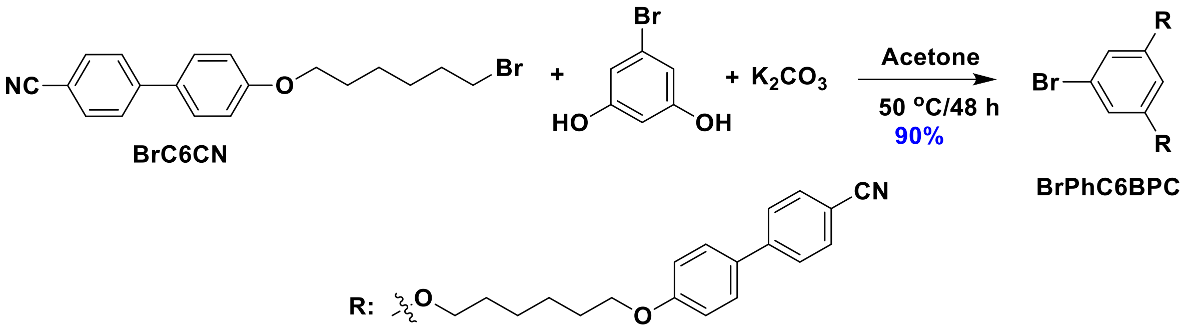

To a 100 mL flask were added **BrC6CN** (1.8 g, 5 mmol, 2.5 equiv.), 5-bromobenzene-1,3-diol (380 mg, 2 mmol, 1 equiv.), and potassium carbonate (840 mg, 6 mmol, 3 equiv.). The mixture was degassed by three cycles of vacuum-nitrogen purging and 50 mL of acetone was injected. The mixture was stirred at 50 °C for 24 h under nitrogen atmosphere. After completion of reaction, 20 mL of water was added to the reaction mixture and extracted with DCM (3 × 50 mL). The combined organic layers were dried with anhydrous magnesium sulfate. The organic solvent was removed under reduced pressure and the crude product was purified by silica gel column chromatography. DCM/Hexane=1/2 was used as eluent to afford **BrPhC6BPC** as a white solid.

Yield: 90% (1.3 g). **R_f**: 0.45 (25% DCM/hexanes on silica gel). **Mp**: 133 °C. **^{1}H NMR (400 MHz, $CDCl_3$) δ (ppm)**: 7.76 – 7.63 (m, 8H), 7.58 – 7.53 (m, 4H), 7.02 (dd, *J* = 8.9, 2.5 Hz, 4H), 6.67 (d, *J* = 2.2 Hz, 2H), 6.39 (t, *J* = 2.2 Hz, 1H), 4.05 (q, *J* = 6.3 Hz, 8H), 3.95 (t, *J* = 6.3 Hz, 8H), 1.97 – 1.75 (m, 0H), 1.59 (dp, *J* = 16.1, 3.6, 3.0 Hz, 8H). **^{13}C NMR (101 MHz, $CDCl_3$) δ (ppm)**: 160.7, 159.7, 145.2, 132.6, 131.4, 128.3, 127.1, 122.9, 119.1, 115.1, 110.2, 110.1, 100.6, 68.1, 29.2, 25.9. **HRMS (ESI-MS) $[M+NH_4]^+$ Calculated**: ($C_{44}H_{47}{}^{79}BrN_3O_4$); 760.2744; **Found**: 760.2741.

4',4'''-((((5-(4,4,5,5-tetramethyl-1,3,2-dioxaborolan-2-yl)-1,3-phenylene)bis(oxy))bis(hexane-6,1-diyl))bis(oxy))bis([1,1'-biphenyl]-4-carbonitrile) (BpinPhC6BPC)

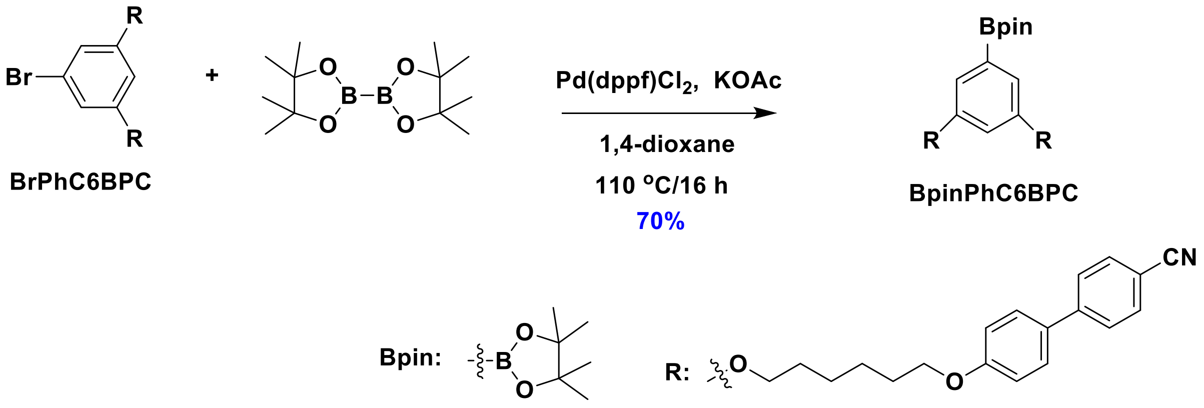

To a 100 mL flask were added **BrPhC6BPC** (1.5 g, 2 mmol, 1 equiv.), bis(pinacolato)diboron (750 mg, 3 mmol, 1.5 equiv.), Pd(dppf)Cl_2 (150 mg, 0.2 mmol, 0.1 equiv.) and potassium acetate (600 mg, 6 mmol, 3 equiv.). The mixture was degassed by three cycles of vacuum-nitrogen purging and 20 mL of 1,4-dioxane was injected. The mixture was stirred at 110 °C for 16 h under nitrogen atmosphere. After completion of reaction, 20 mL of water was added to the reaction mixture and extracted with DCM (3 × 50 mL). The combined organic layers were dried with anhydrous magnesium sulfate. The organic solvent was removed under reduced pressure and the crude product was purified by silica gel column chromatography. DCM was used as eluent to afford **BpinPhC6BPC** as a white solid.

Yield: 70% (1.1 g). **R$_f$**: 0.55 (DCM on silica gel). **Mp**: 103 °C. **^{1}H NMR (400 MHz, $CDCl_3$) δ (ppm)**: 7.77 – 7.62 (m, 8H), 7.55 (d, J = 8.8 Hz, 4H), 7.02 (d, *J* = 8.8 Hz, 4H), 6.96 (d, *J* = 2.4 Hz, 2H), 6.59 (s, 1H), 4.04 (t, *J* = 6.6 Hz, 4H), 4.00 (t, *J* = 6.3 Hz, 4H), 1.85 (dt, *J* = 13.9, 6.7 Hz, 8H), 1.57 (p, *J* = 3.6 Hz, 8H), 1.36 (s, 12H). **^{13}C NMR (101 MHz, $CDCl_3$) δ (ppm)**: 159.9, 159.7, 145.3, 132.6, 131.3, 128.3, 127.1, 119.1, 115.1, 112.3, 110.0, 110.0, 105.2, 83.9, 68.0, 67.8, 29.3, 29.2, 25.9, 25.8, 24.9. **HRMS (ESI-MS) [M+H]$^+$ Calculated**: ($C_{50}H_{56}BN_2O_6$); 791.4153; **Found**: 791.4234.

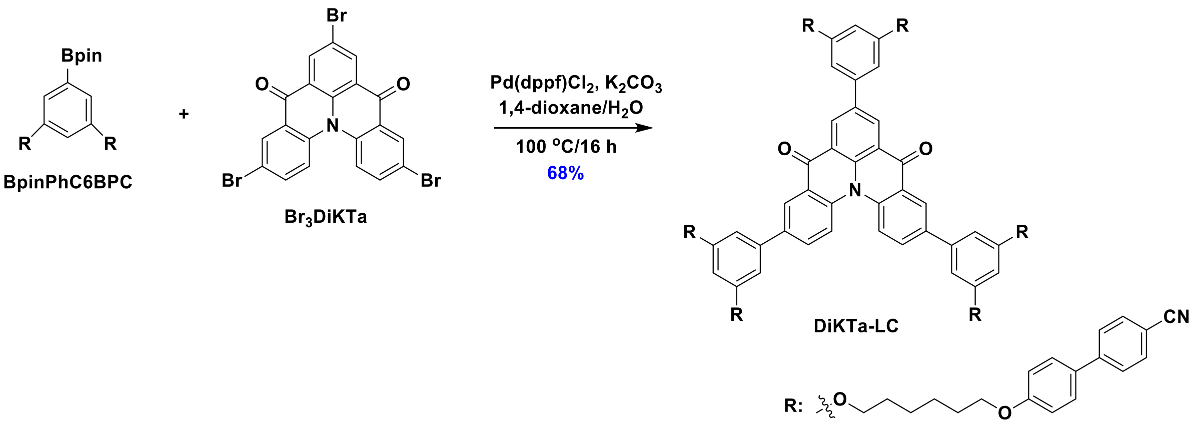

To a 250 mL flask were added **Br$_3$DiKTa** (270 mg, 0.5 mmol, 1 equiv.), **BpinPhC6BPC** (1.6 g, 2 mmol, 4 equiv.), Pd(dppf)Cl_2 (75 mg, 0.1 mmol, 0.2 equiv.), potassium carbonate (420 mg, 3 mmol, 6 equiv.), 20 mL of 1,4-dioxane and 4 mL of water. The mixture was degassed by three cycles of freeze-pump-thaw and stirred at 110 °C for 16 h under nitrogen atmosphere. After completion of reaction, 20 mL of water was added to the reaction mixture and extracted with DCM (3 × 50 mL). The combined organic layers were dried with anhydrous magnesium sulfate. The organic solvent was removed under reduced pressure and the crude product was purified by silica gel column chromatography, DCM/Hex = 1/3 was used as eluent to afford **DiKTa-LC** as a yellow solid.

Yield: 68%. **R_f** = 0.52 (50% DCM/Hexane). **Mp:** 115 °C. **^{1}H NMR (400 MHz, CDCl$_3$) δ (ppm):** 9.02 (s, 2H), 8.75 (d, *J* = 2.3 Hz, 2H), 8.24 (d, *J* = 8.9 Hz, 2H), 7.97 (dd, J = 8.9, 2.3 Hz, 2H), 7.74 – 7.59 (m, 24H), 7.57 – 7.48 (m, 12H), 7.01 (dd, *J* = 8.9, 2.4 Hz, 12H), 6.96 (d, *J* = 2.2 Hz, 2H), 6.90 (d, *J* = 2.2 Hz, 4H), 6.57 (d, *J* = 2.0 Hz, 3H), 4.11 (t, *J* = 6.3 Hz, 12H), 4.06 (t, *J* = 6.5 Hz, 12H), 1.95 – 1.88 (m, 24H), 1.63 (s, 24H). **^{13}C NMR δ (ppm):** 178.6, 161.0, 160.9, 159.7, 145.2, 140.8, 138.8, 138.2, 132.6, 132.5, 131.5, 131.3, 131.2, 131.1, 128.4, 128.3, 127.0, 126.5, 125.6, 123.8, 120.8, 119.1, 115.1, 110.1, 109.9, 105.8, 100.9, 68.0, 29.2, 25.9. **HRMS (MALDI-TOF): [M+Na]$^+$ Calculated** [$C_{152}H_{137}N_7O_{14}Na$]$^+$: 2308.0155, found 2308.0296. **Elemental analysis: Calcd for $C_{152}H_{137}N_7O_{14}$**: C, 79.87; H, 6.04; N, 4.29. **Found**: C, 79.45; H, 6.16; N, 4.34. **HPLC** 10% THF/MeCN, 1.0 mL min^{-1}, 300 nm; tr (96.8 %) = 16.4 min.

Liquid crystal characteristics studies

Materials and Procedures

A polarizing optical microscope Olympus BX50 equipped with a variable temperature sample holder LTS350 (control unit: TP39 and LNP, ΔT = ± 1 K) by Linkam Scientific was used to investigate the mesomorphic properties of our compounds. Micrographs were taken with a Zeiss Axiocam 105 color camera module and the software ZEN core. The samples were investigated on regular glass slides and in polyimide coated cells. DSC measurements were performed on a DSC822^e by Mettler Toledo in

standardized 40 μL aluminum crucibles and evaluated with the software STARe 14.0. Phase transition temperatures are given as onsets of the corresponding peaks. For Xray diffraction studies, the samples were sealed in glass capillaries supplied by Hilgenberg GmbH (external diameter of 0.7 mm, wall thickness 0.01 mm). Two dimensional Xray diffraction (2D-WAXS and 2D-SAXS) studies were carried out on a Bruker AXS Nanostar C equipped with a ceramic tube generator (CuK_α radiation, λ = 1.5405 Å, 1500 W) and a Bruker Vantec 500 detector. The diffractograms were processed using SAXS software and calibrated to the diffraction pattern of silver behenate at 25 °C. One dimensional Xray diffraction (1D-SAXS) studies were carried out on a SAXSess by Anton Parr GmbH (CuK_α radiation) equipped with a CMOS detector (Dectris, Mythen 1K) and a variable temperature sample holder (TCS 120). The diffractograms were processed using SAXSquant 3.5 software and calibrated to the diffraction pattern of cholesteryl palmitate at 25 °C.

DSC studies:

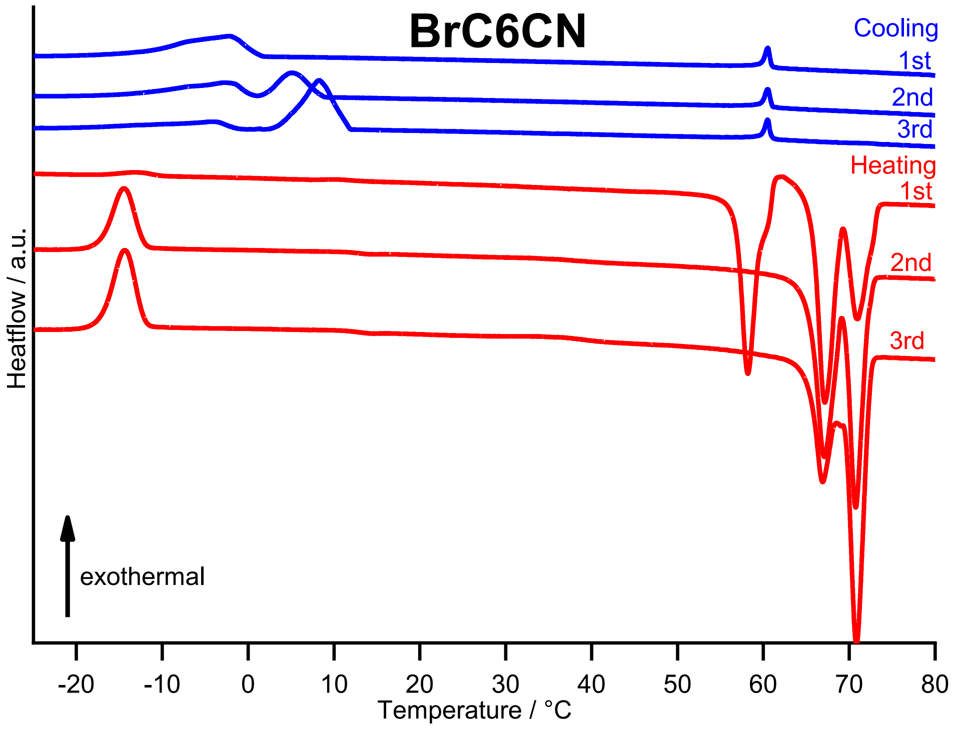

Figure **S3.** DSC thermogram of **BrC6CN** recorded with heating/ cooling rates of 10 K min^{-1}.

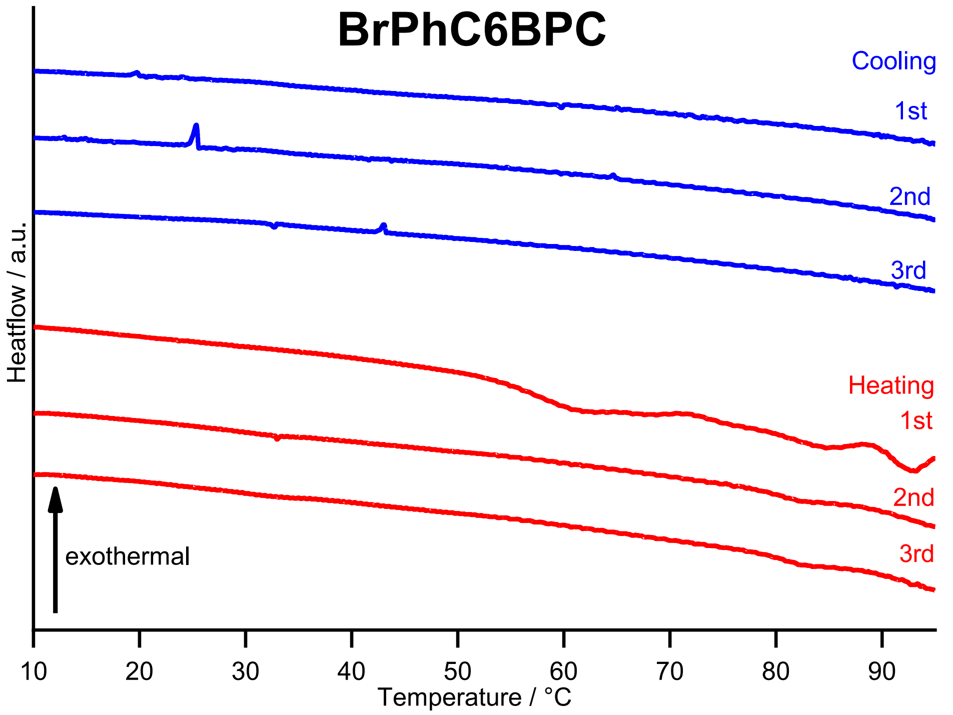

Figure **S4.** DSC thermogram of **BrPhC6BPC** recorded with heating/ cooling rates of 10 K min^{-1}.

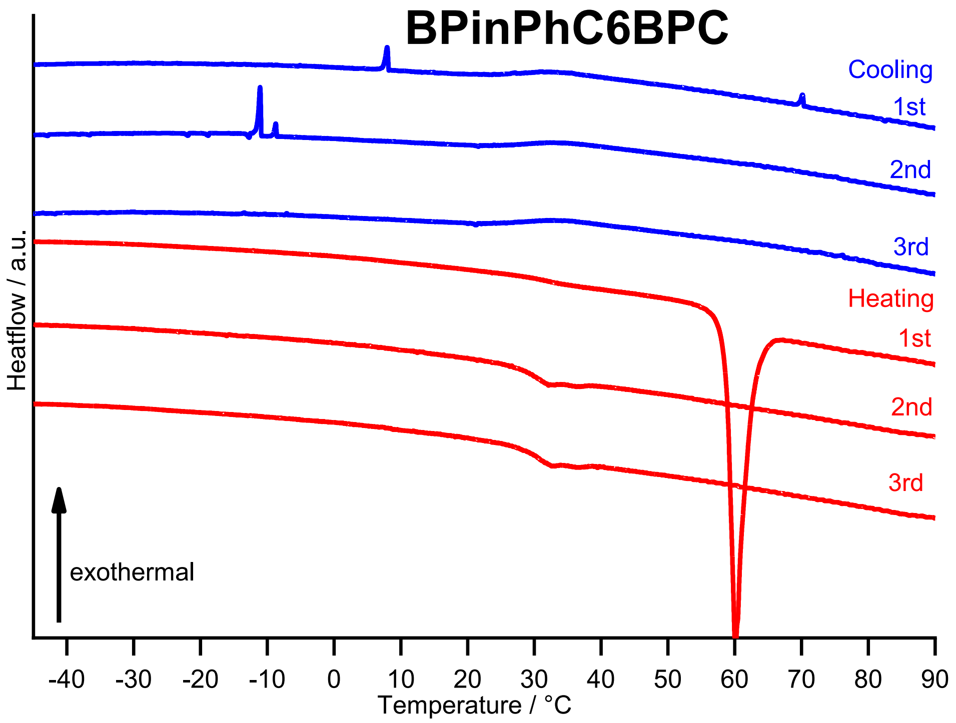

Figure **S5.** DSC thermogram of **BPinPhC6BPC** recorded with heating/ cooling rates of 10 K min^{-1}.

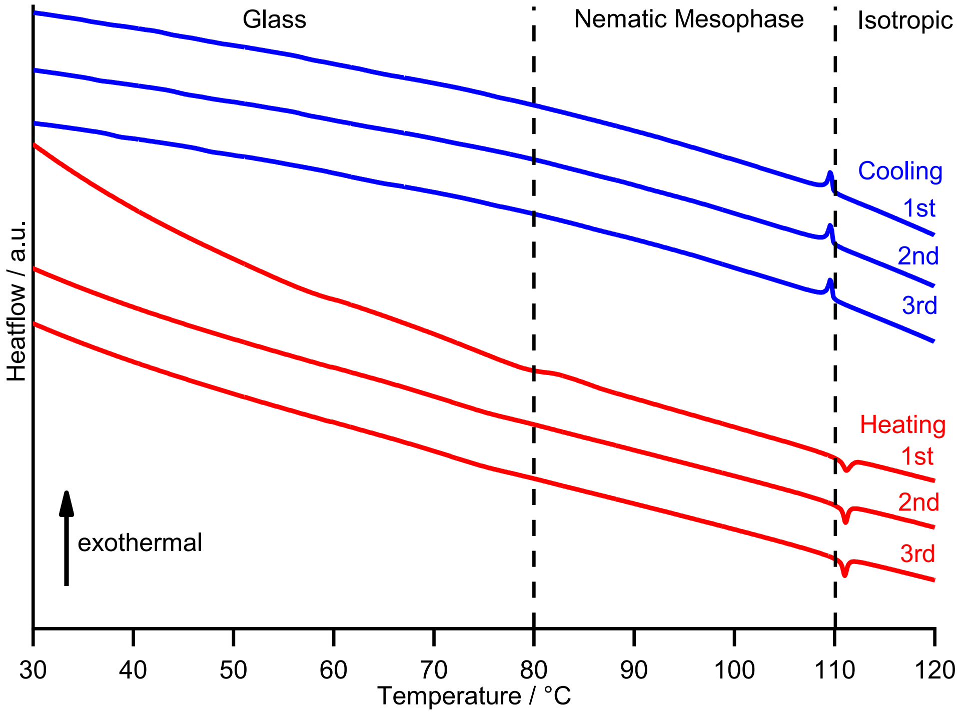

Figure **S6.** DSC thermogram of **DiKTa-LC** recorded with heating/ cooling rates of 10 K min^{-1}. Clearing points and enthalpies of **DiKTa-LC**: 110 °C, -1.89 kJ mol^{-1} (2nd heating cycle), 110 °C, 1.42 kJ mol^{-1} (2nd cooling cycle).

2D-WAXS and SAXS studies:

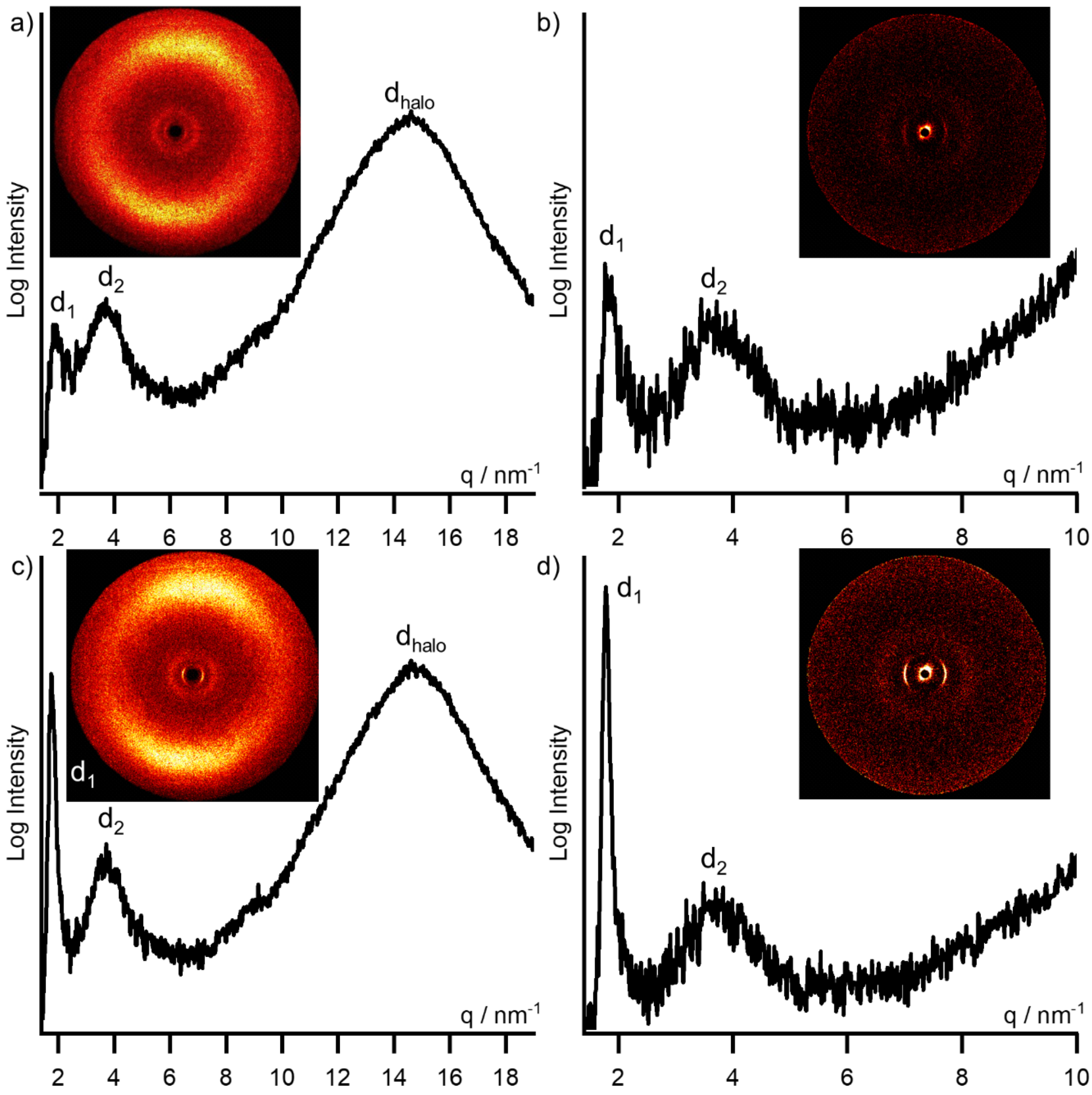

Figure **S7**. Xray diffractograms and 2D diffraction patterns (insets) of **DiKTa-LC** recorded on a Bruker Nanostar: a) WAXS at 97 °C (nematic Phase), b) SAXS at 97 °C (nematic Phase), c) WAXS at 69 °C (glass phase), d) SAXS at 69 °C (glass phase).

1D-SAXS studies:

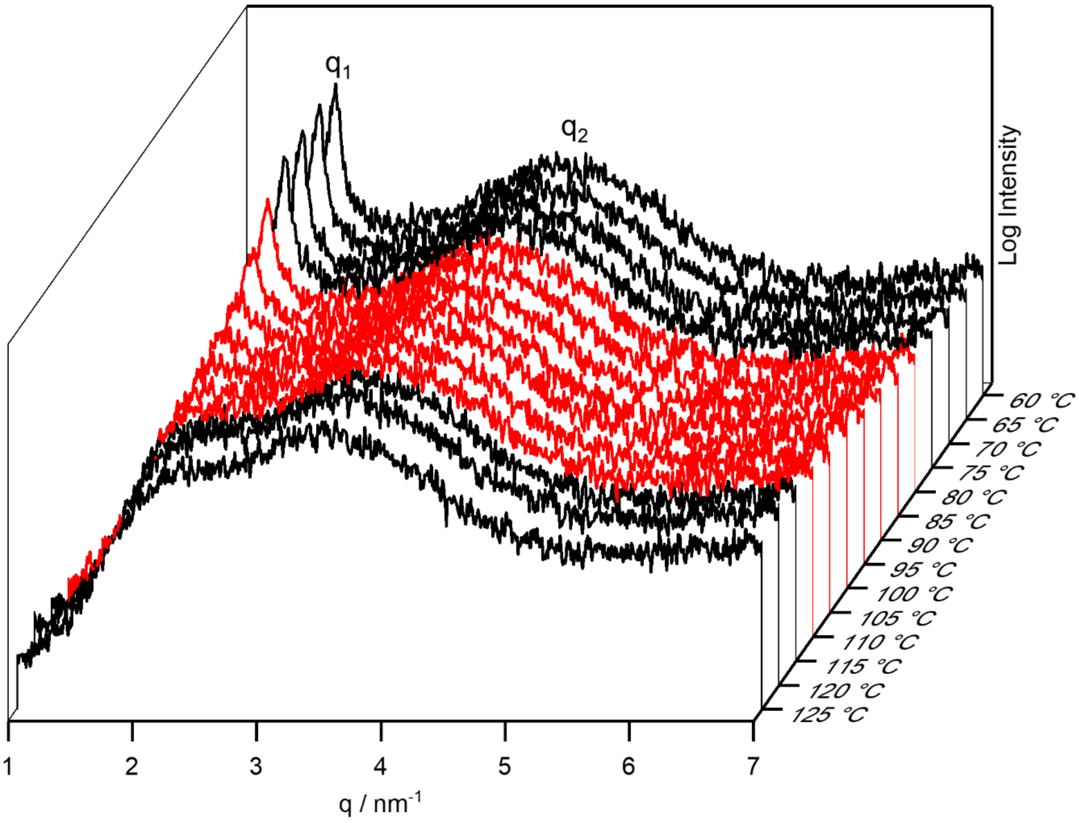

Figure **S8**. Temperature dependent Xray diffraction patterns of **DiKTa-LC** recorded on a SAXSess (q_{min} = 1 nm^{-1}, d_{max} = 62.8 Å). Traces at mesophase temperature are given in red.

Table **S1**. Scattering vectors and corresponding distances of **DiKTa-LC** determined from the SAXS diffractograms.

Temperature / °C	q_1 / nm^{-1}	q_2 / nm^{-1}	d_1 / Å	d_2 / Å
60	1.78	3.61	35.3	17.4
65	1.78	3.60	35.3	17.5
70	1.78	3.60	35.3	17.5
75	1.78	3.61	35.3	17.4
80	1.78	3.61	35.3	17.4
85	1.78	3.60	35.3	17.5
90	1.80	3.61	34.9	17.4
95	1.80	3.60	34.9	17.5
100	1.83	3.60	34.3	17.5
105	1.90	3.60	33.1	17.5
110	1.96	3.60	32.1	17.5
115	2.06	3.60	30.5	17.5
120	2.30	3.60	27.3	17.5
125	2.40	3.59	26.2	17.5

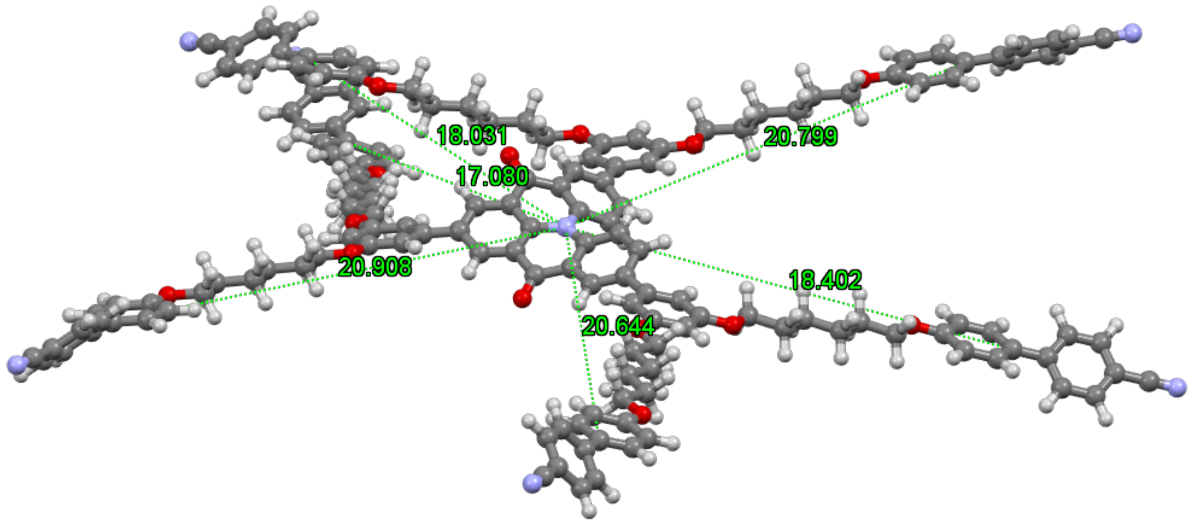

Figure **S9**. Measured distances (Å) between the aromatic core and the cyanobiphenyl groups of the DFT-optimized **DiKTa-LC** structure (average of the 6 distances: d_{intra} = 19.3 Å). For computational details please refer to page S3.

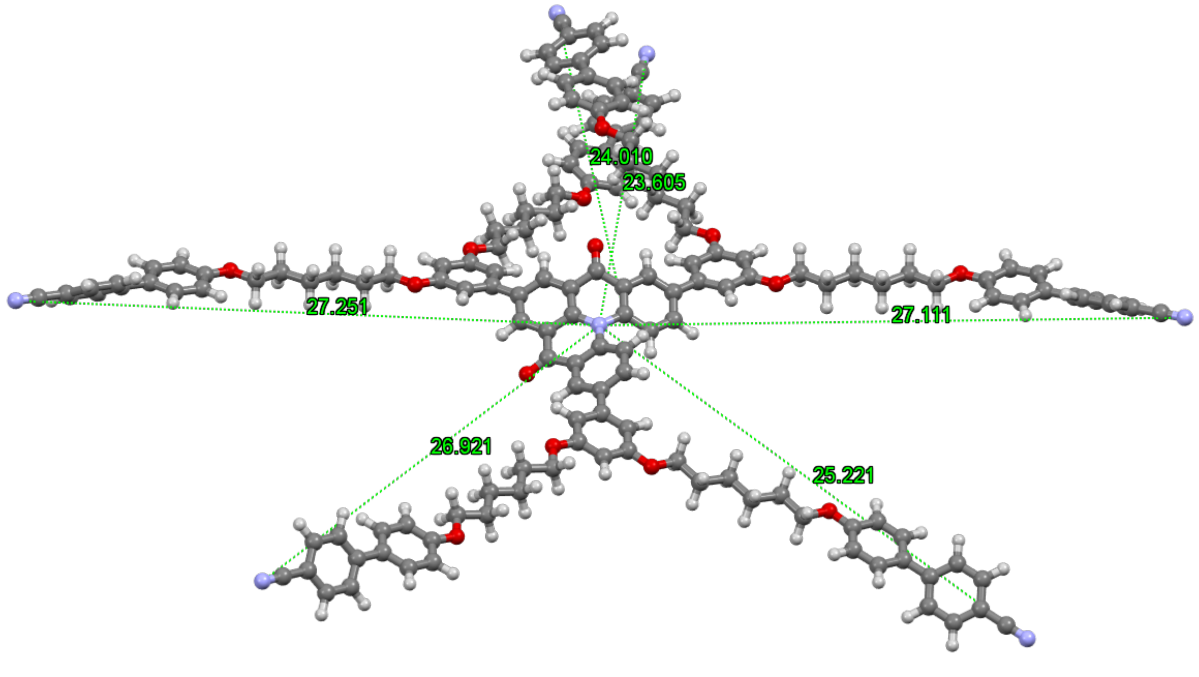

Figure **S10**. Measured radii (Å) of the DFT-optimized **DiKTa-LC** structure ($r_{average}$ = 25.8 Å, d = 2*r = 51.6 Å). For computational details please refer to page S3.

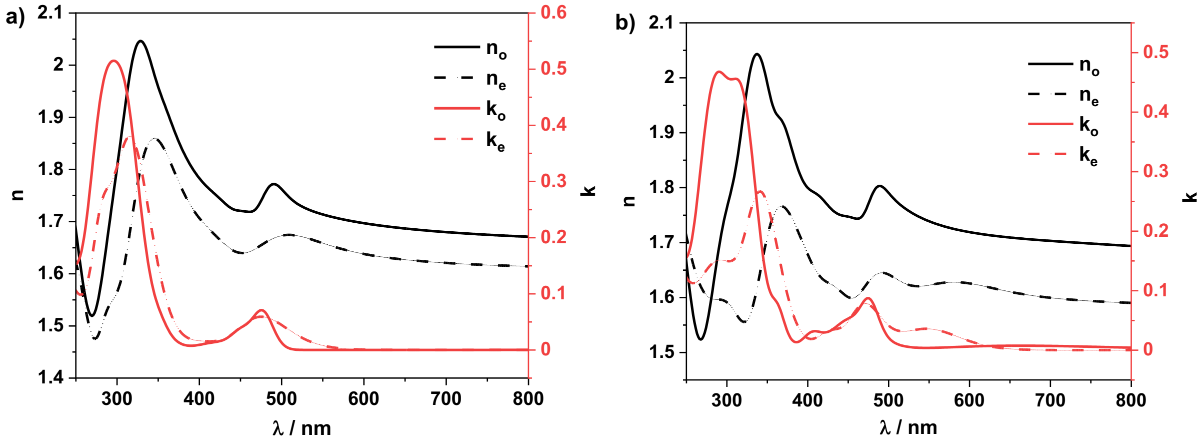

Figure **S11**. Anisotropic refractive indices and extinction coefficients of **DiKTa-LC** neat film a) pristine and b) annealed. The black solid and dashed lines show the real component of the refractive indices in the horizontal and vertical directions (n_o and n_e), respectively. The red solid and dashed lines show the extinction coefficients in the horizontal and vertical directions (k_o and k_e), respectively. The VASE analysis shows that, for a pristine film, the values of a for absorption at 290 nm and 475 nm (absorption bands of the cyano-biphenyl moieties and **DiKTa** core, respectively) are 0.20 and 0.29, respectively. These values indicate 80%- and 71%-horizontal orientation, respectively. For the annealed film, the TDM at 293 nm shows 94%-horizontal orientation (a = 0.06), and at 474 nm the TDM retains a 69%-horizontal orientation (a = 0.31).

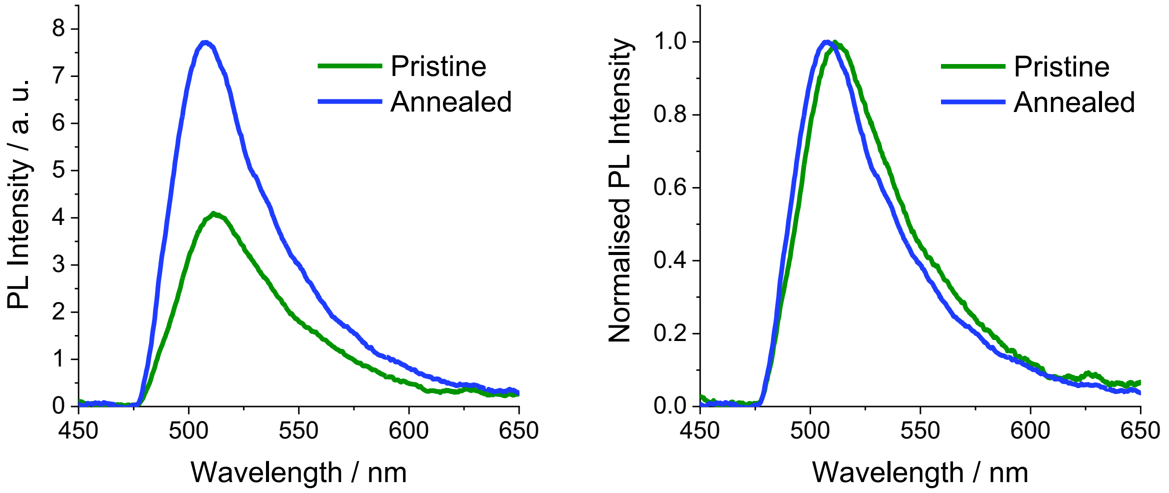

Figure **S12**. PL spectrum of the neat films of **DiKTa-LC** measured at 0° in the ARPL setup. The annealed film exhibited a higher PL intensity and a slightly blue-shifted peak wavelength (508 nm) with respect to the pristine film (511 nm).

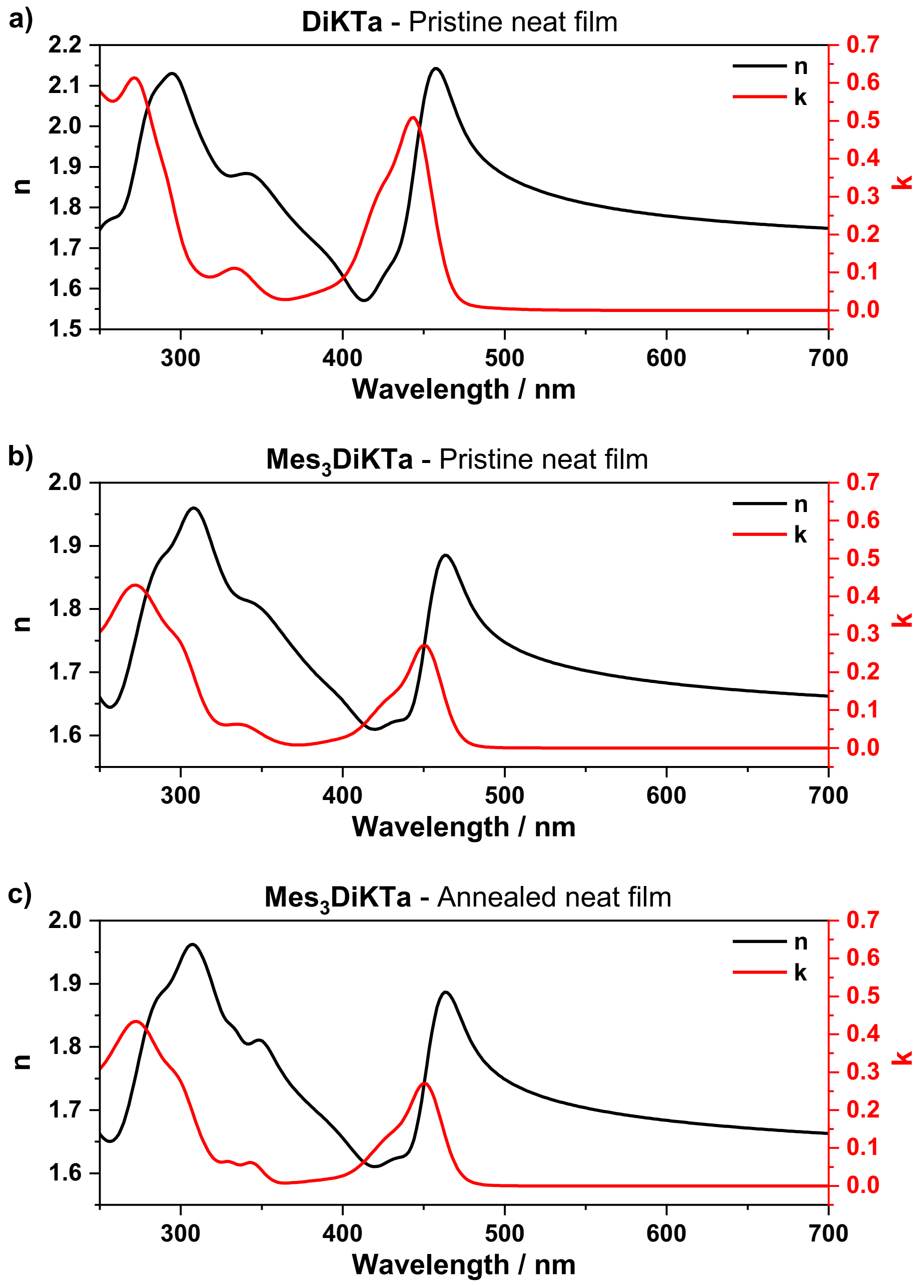

Figure **S13**. Isotropic refractive indices of pristine films of **DiKTa** (a) and **Mes₃DiKTa** (b), and of annealed films of **Mes₃DiKTa**. Anisotropic fits to the ellipsometry data did not yield significant

differences between the ordinary and extraordinary components, so the data was fitted to an isotropic model.

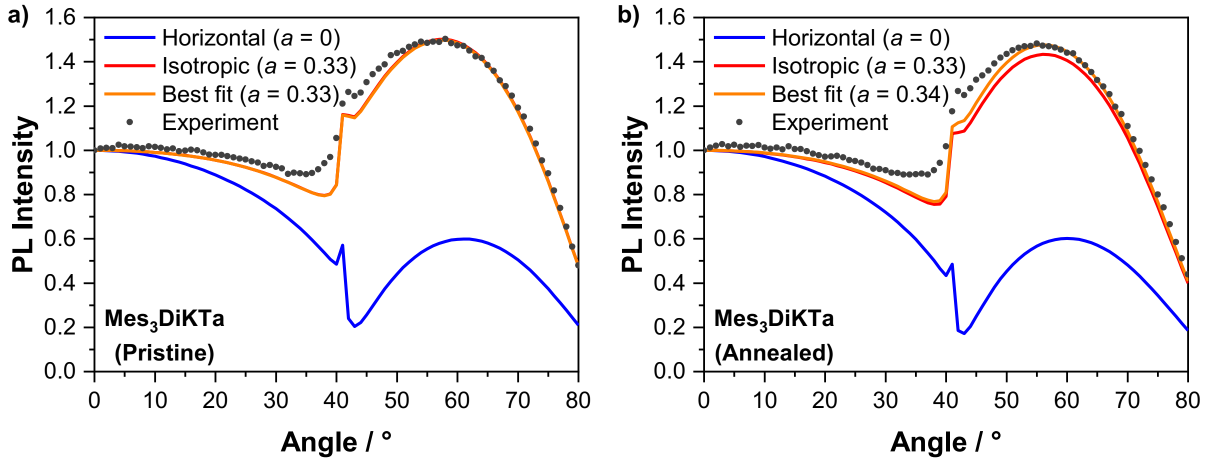

Figure **S14**. ARPL patterns of the neat films of **Mes$_3$DiKTa**.

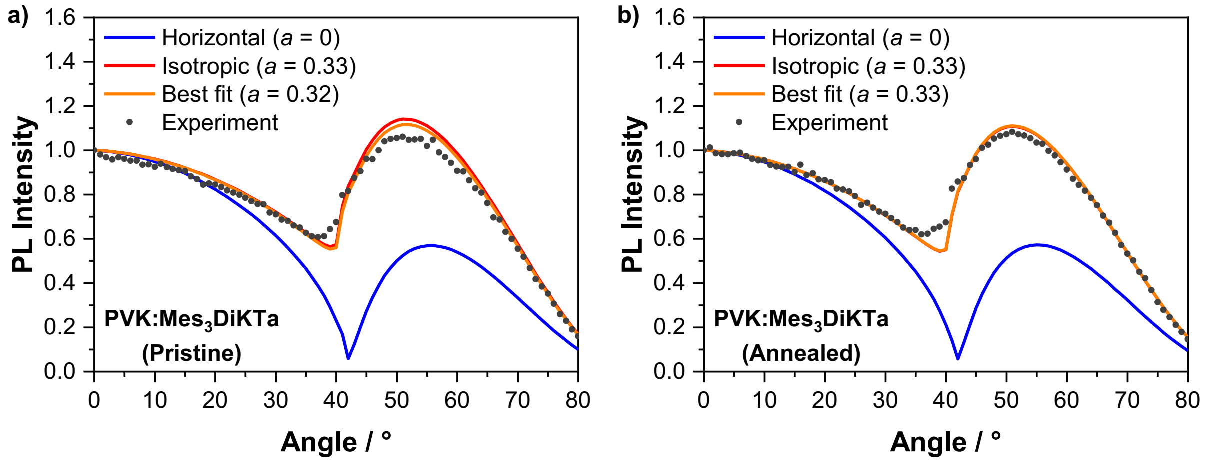

Figure **S15**. ARPL patterns of the films of **Mes$_3$DiKTa** doped at 10 wt% in PVK.

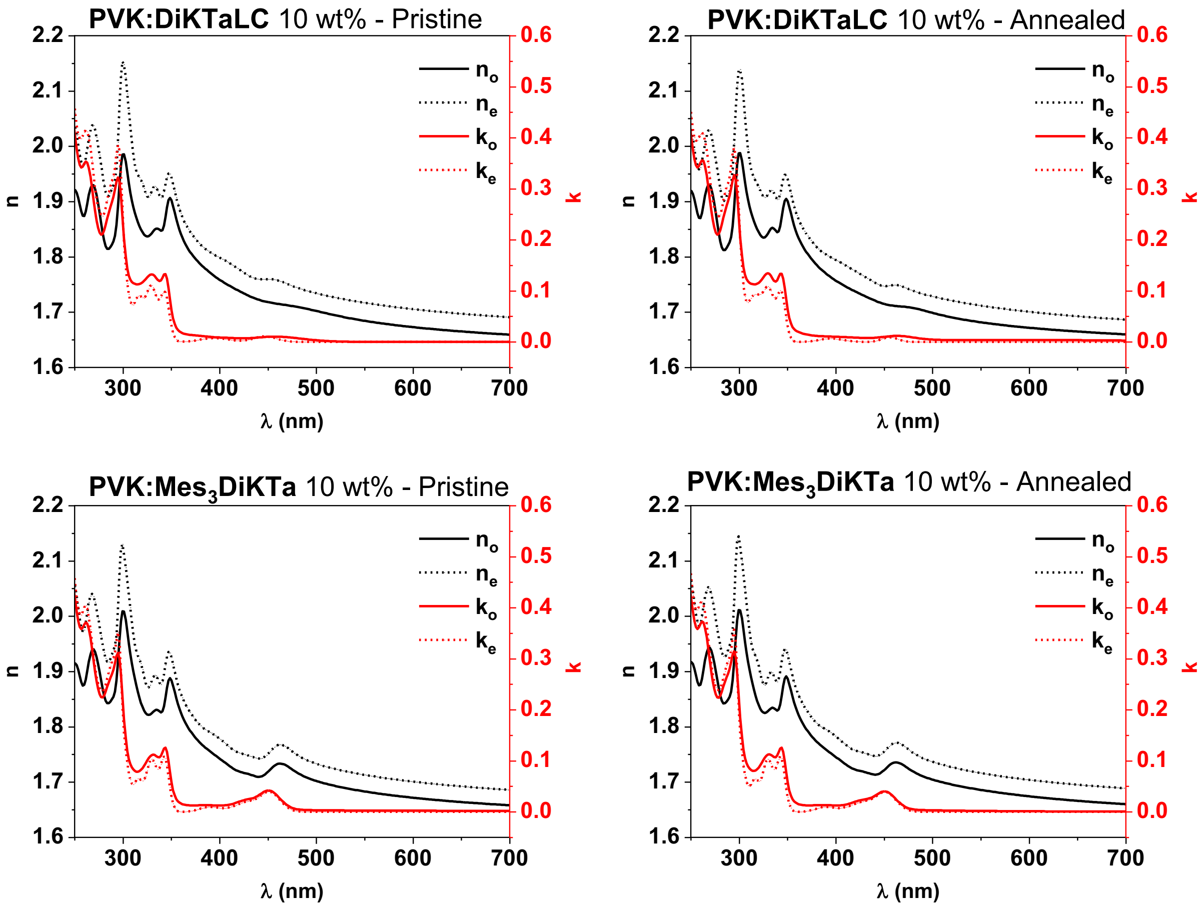

Figure **S16**. Anisotropic refractive indices and extinction coefficients of doped films consisting of **DiKTa-LC** and **Mes3DiKTa** in PVK at a 10 wt% ratio. The black solid and dashed lines show the real component of the refractive indices in the horizontal and vertical directions (n_o and n_e), respectively. The red solid and dashed lines show the extinction coefficients in the horizontal and vertical directions (k_o and k_e), respectively. The consistent difference between n_o and n_e across these measurements can be ascribed to the birefringence of PVK.[17]

Table **S2**. Fitted values of the anisotropy factor (*a*) and film thickness for each dataset from ARPL data.

Emitter	Condition	Neat film		Doped film	
		Fitted *a*	Fitted thickness (nm)	Fitted *a*	Fitted thickness (nm)
**Mes3DiKTa**	Pristine	0.33	61	0.32	34
	Annealed	0.34	56	0.33	31
**DiKTa-LC**	Pristine	0.28	47	0.28	30
	Annealed	0.28	48	0.29	30

OLED fabrication and characterization

The OLED devices were fabricated using a bottom-emitting architecture. A pre-patterned indium tin oxide (ITO) glass substrate with a sheet resistance of 15 Ω square-1 was pre-cleaned carefully with detergent and deionized water and then exposed to UV-ozone for 15 min. PEDOT:PSS was spin-coated onto the clean ITO substrate as the hole-injection layer, followed by thermal treatment under 120 °C for 30 min. Then a 10 mg/mL chlorobenzene solution of our dendrimers was spin-coated to form a 35-45 nm thick emissive layer (EML) and annealed at 120 °C for 10 min to remove residual solvent before transfer to the vacuum chamber. A 40 nm-thick electron-transporting layer (ETL) of Tm3PyPB was then vacuum deposited at a rate of 1 Å/s, which was controlled in situ using quartz crystal monitors. The electron injection layer LiF was deposited at a rate of 0.1 Å/s while the Al cathode was deposited at a rate of 10 Å/s through the shadow mask defining the top electrode. The spatial overlap of the anode and cathode electrodes determined the active area of the OLED, which was estimated to be 4 mm^2. Electroluminescence (EL), CIE color coordinates, and spectra were obtained via a Spectrascan PR655 photometer, and the luminance-current-voltage characteristics were determined with a computer-controlled Keithley 2400 Source meter. EQE was calculated from the current density, luminance, and EL spectrum, assuming Lambertian emission distribution.

Compound Characterization

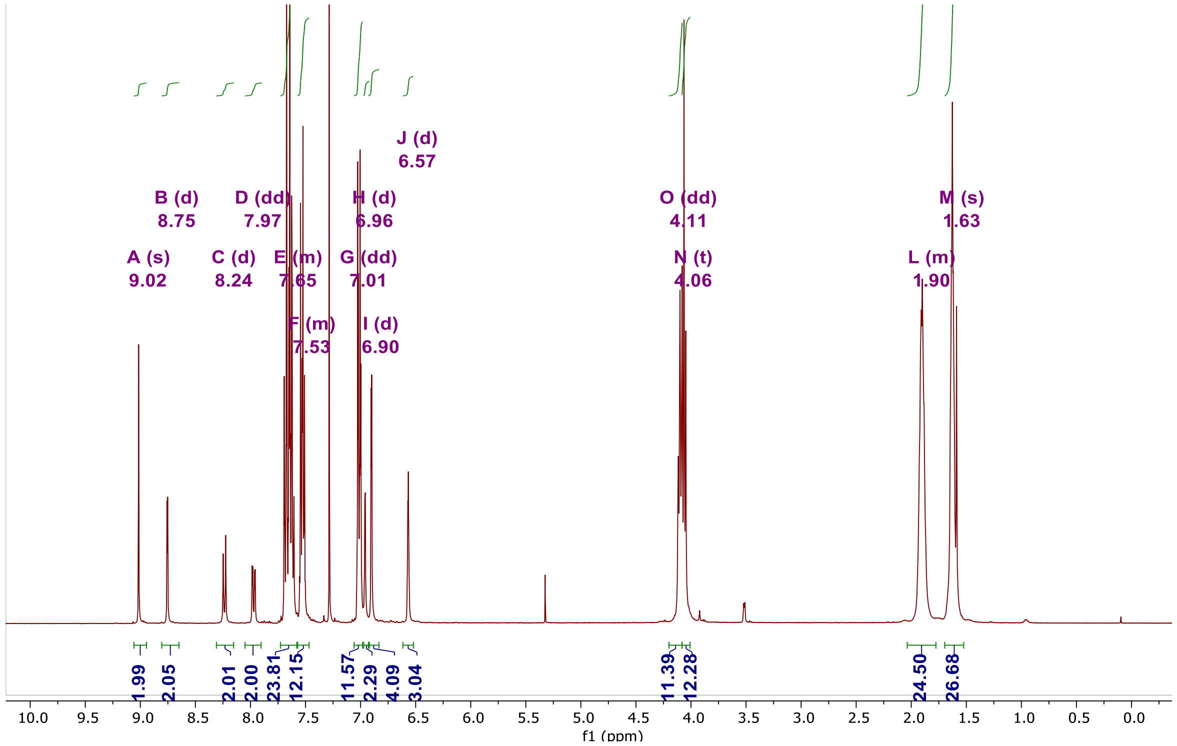

Figure **S17**. ^{1}H NMR of **DiKTa-LC** in $CDCl_3$

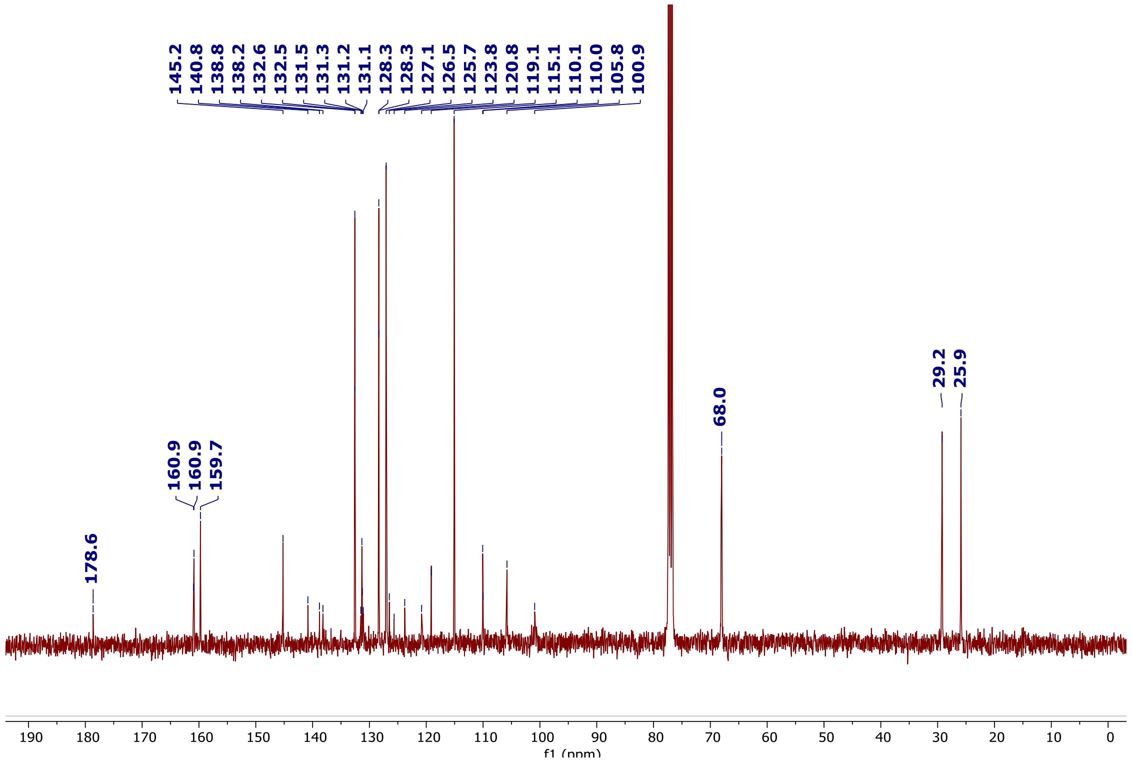

Figure **S18**. ^{13}C NMR of **DiKTa-LC** in $CDCl_3$

School of Chemistry Mass Spectrometry Service

**SampleID**						**Submitter**		
**Sample Description**								
**Analysis Name**	D:\Data\stuartwarriner\DikaLC_g.d					**Supervisor**		
**Method**	OMDirectFeBox_MSmethod.m					**Acquisition Date**	22/02/2021 09:14:08	
**Instrument**	maXis impact	**Source Type**	ESI	**Ion Polarity**	Positive	**Scan Begin** 50 m/z	**Scan End**	3000 m/z

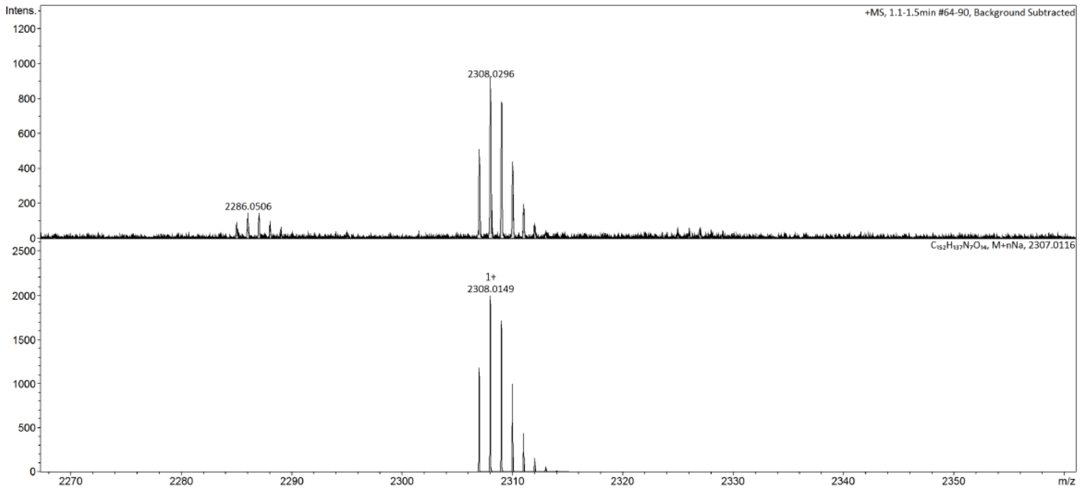

Bruker Compass DataAnalysis 4.3 | Analysis Name | DikaLC_g.d | 22/02/2021 10:15:05 | 1 of 1

Figure **S19**. HRMS of **DiKTa-LC**

Elemental Analysis Service Request Form

Researcher name **Dongyang Chen**

Researcher email **dc217@st-andrews.ac.uk**

NOTE: Please submit ca. 10 mg of sample

**Sample reference number**	dc-III 18
**Name of Compound**	DiKTaLC
**Molecular formula**	$C_{152}H_{137}N_7O_{14}$
**Stability**	stable
**Hazards**	low hazard
**Other Remarks**	

Analysis type:

Single ☐ **Duplicate**√☐ **Triplicate**☐

Analysis Result:

Element	Expected %	Found (1)	Found (2)	Found (3)
Carbon	79.87	79.44	79.47	
Hydrogen	6.04	6.17	6.16	
Nitrogen	4.29	4.30	4.37	
Oxygen				

Authorising Signature:

**Date completed**	21.05.21
**Signature**	J-P C
**comments**	

Figure **S20**. Elemental analysis of **DiKTa-LC**

SHIMADZU LabSolutions

Analysis Report

<Sample Information>

Sample Name	: dc-C6CO		
Sample ID	: THF/AcN=10/90		
Data Filename	: 19042021-THF/AcN DCC6CNDiKTa		
Method Filename	: THF/AcN 10%		
Batch Filename	: DCC6CNDiKTa.lcb		
Vial #	: 1-19	Sample Type	: Unknown
Injection Volume	: 10 uL		
Date Acquired	: 19/04/2021 21:03:25 21:03:25	Acquired by	: ezc-7
Date Processed	: 19/04/2021 21:33:28 21:33:28	Processed by	: ezc-7

<Chromatogram>

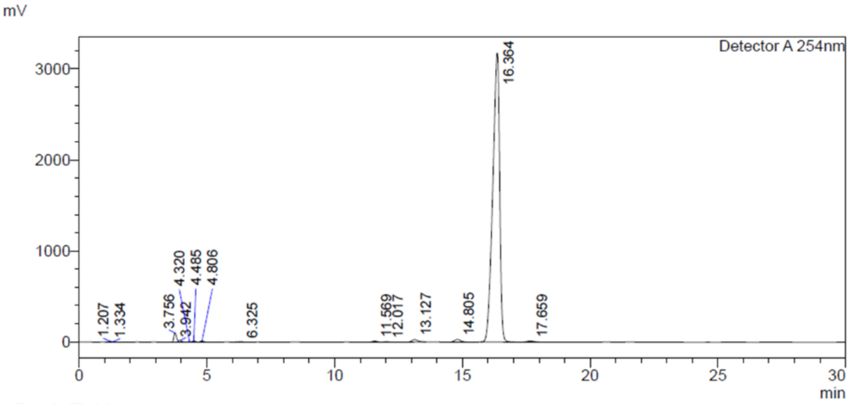

<Peak Table>

Detector A 254nm

Peak#	Ret. Time	Area	Height	Conc.	Unit	Mark	Name
1	1.207	58705	8199	0.097			
2	1.334	21706	4127	0.036		V	
3	3.756	738453	100717	1.219			
4	3.942	129583	16779	0.214		V	
5	4.320	37704	5264	0.062		V	
6	4.485	104153	12833	0.172		V	
7	4.806	103467	10696	0.171		V	
8	6.325	63977	4440	0.106			
9	11.569	128581	10054	0.212			
10	12.017	56687	4345	0.094		V	
11	13.127	412097	26334	0.680			
12	14.805	478776	28213	0.790			
13	16.364	58067721	3172628	96.832			
14	17.659	191785	9181	0.317			
Total		60593394	3413811				

Figure **S21**. HPLC report of **DiKTa-LC**

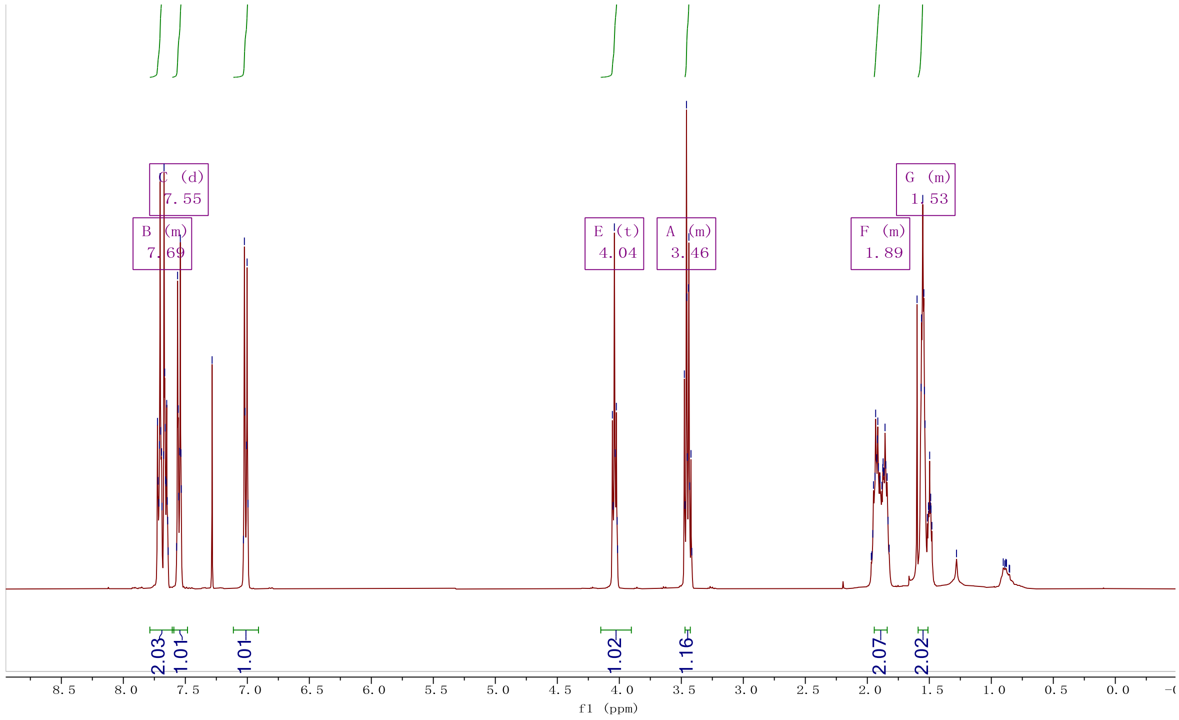

Figure **S22**. ^{1}H NMR of **BrC6CN** in $CDCl_3$

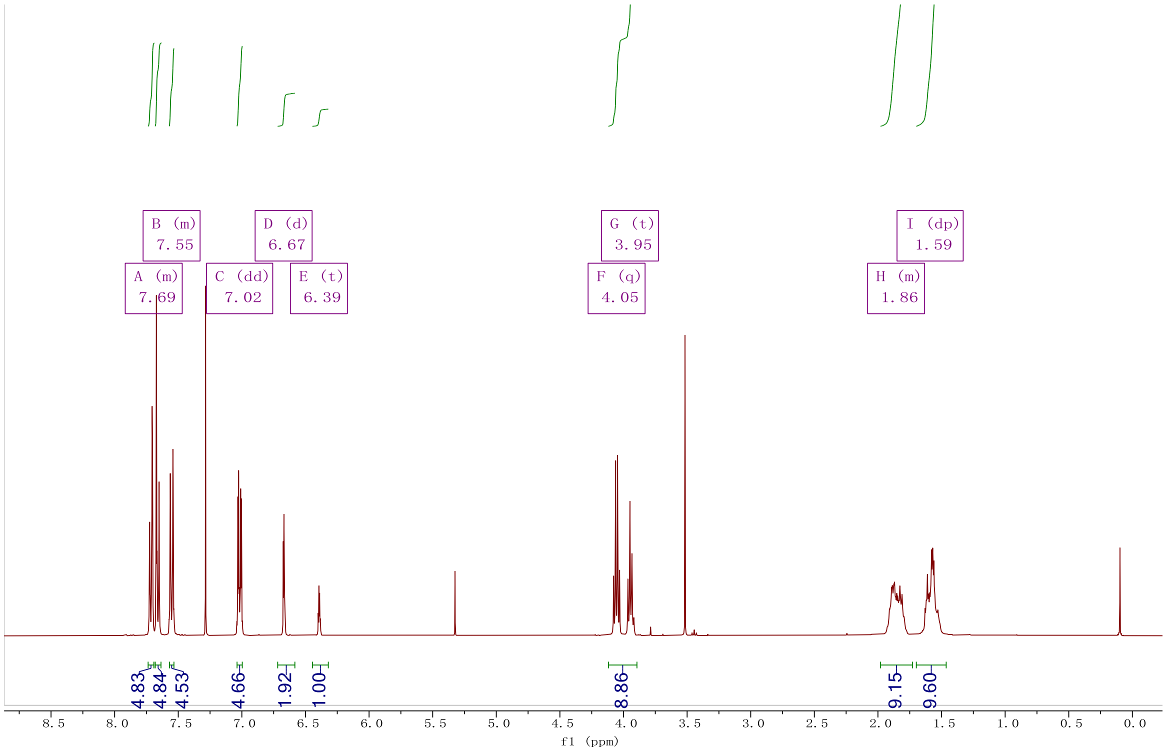

Figure **S23**. ^{1}H NMR of **BrPhC6BCP** in $CDCl_3$

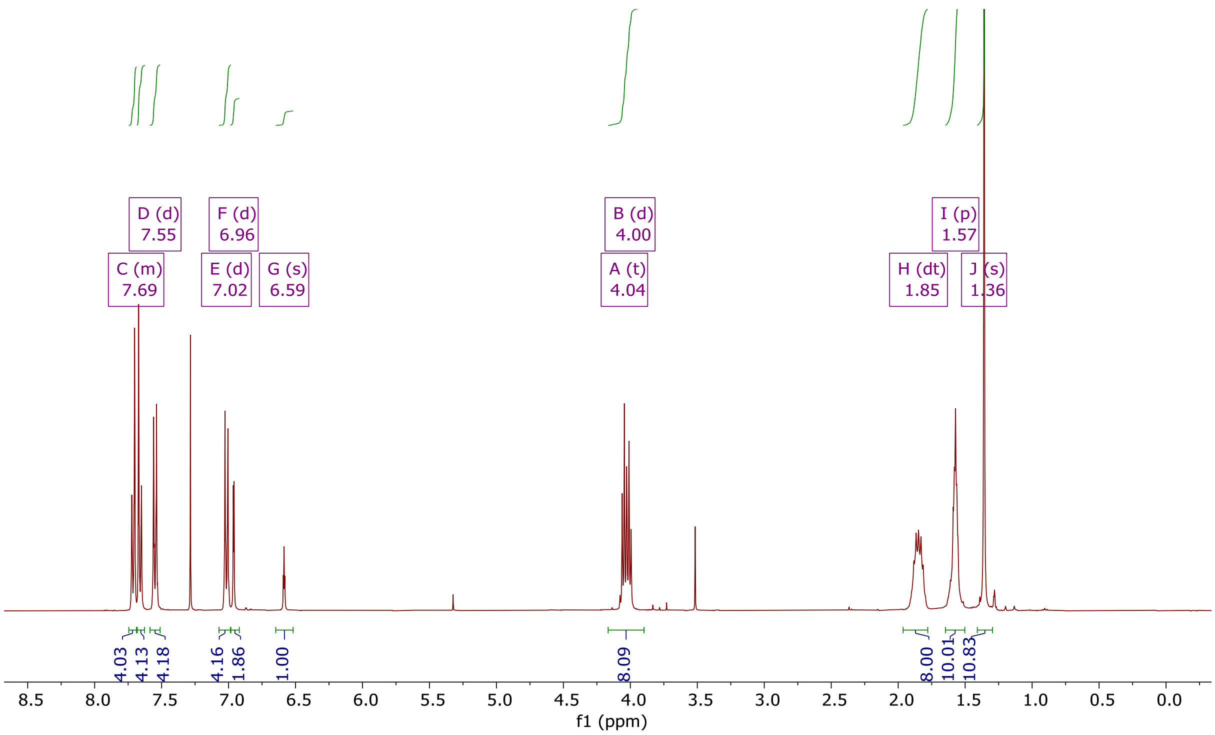

Figure **S24**. ^{1}H NMR of **BpinPhC6BCP** in $CDCl_3$

References

(1) Frisch, M. J.; Trucks, G. W.; Schlegel, H. B.; Scuseria, G. E.; Robb, M. A.; Cheeseman, J. R.; Scalmani, G.; Barone, V.; Mennucci, B.; Petersson, G. A.; Nakatsuji, H.; Caricato, M.; Li, X.; Hratchian, H. P.; Izmaylov, A. F.; Bloino, J.; Zheng, G.; Sonnenberg, J. L.; Had, M.; Fox, D. J. Gaussian 09, Revis. D.01. Wallingford, CT. 2009.

(2) TURBOMOLE V6.5. TURBOMOLE GmbH, since 2007. https://doi.org/http://www.turbomole.com.

(3) Adamo, C.; Barone, V. Toward Reliable Density Functional Methods without Adjustable Parameters: The PBE0 Model. *J. Chem. Phys.* **1999**, *110* (13), 6158–6170. https://doi.org/10.1063/1.478522.

(4) Becke, A. D. Dependence of the Virial Exciton Model on Basis Set and Exact-Exchange Fraction. *J. Chem. Phys.* **2019**, *150* (24), 241101. https://doi.org/10.1063/1.5109675.

(5) R. Dennington, T.Keith;J. Millam, GaussView, Version 5, KS: Semichem Inc.: Shawnee Mission, 2009.

(6) Petersson, G. A.; Bennett, A.; Tensfeldt, T. G.; Al-Laham, M. A.; Shirley, W. A.; Mantzaris, J. A Complete Basis Set Model Chemistry. I. The Total Energies of Closed-Shell Atoms and Hydrides of the First-Row Elements. *J. Chem. Phys.* **1988**, *89* (4), 2193–2218. https://doi.org/10.1063/1.455064.

(7) Momma, K.; Izumi, F. Journal of Applied Crystallography - 2011 - Momma - VESTA 3 for Three-dimensional Visualization of Crystal Volumetric and.Pdf. *J. Appl. Crystallogr.* **2011**, *44*, 1272–1276. https://doi.org/10.1107/S0021889811038970.

(8) Connelly, N. G.; Geiger, W. E. Chemical Redox Agents for Organometallic Chemistry. *Chem. Rev.* **1996**, *96* (2), 877–910. https://doi.org/10.1021/cr940053x.

(9) Cardona, C. M.; Li, W.; Kaifer, A. E.; Stockdale, D.; Bazan, G. C. Electrochemical Considerations for Determining Absolute Frontier Orbital Energy Levels of Conjugated Polymers for Solar Cell Applications. *Adv. Mater.* **2011**, *23* (20), 2367–2371. https://doi.org/10.1002/adma.201004554.

(10) Demas, J. N.; Crosby, G. A. The Measurement of Photoluminescence Quantum Yields. A Review. *J. Phys. Chem.* **1971**, *75* (4), 991–1024.

(11) Greenham, N. C.; Samuel, I. D. W.; Hayes, G. R.; Phillips, R. T.; Kessener, Y. A. R. R.; Moratti, S. C.; Holmes, A. B.; Friend, R. H. Measurement of Absolute Photoluminescence Quantum Efficiencies in Conjugated Polymers. *Chem. Phys. Lett.* **1995**, *241*, 89–96. https://doi.org/10.1016/0009-2614(95)00584-Q.

(12) Archer, E.; Hillebrandt, S.; Keum, C.; Murawski, C.; Murawski, J.; Tenopala-Carmona, F.; Gather, M. C. Accurate Efficiency Measurements of Organic Light-Emitting Diodes via Angle-Resolved Spectroscopy. *Adv. Opt. Mater.* **2021**, *9*, 2000838. https://doi.org/10.1002/adom.202000838.

(13) Kim, N. S.; Kim, D. Y.; Song, J. H.; Suh, M. C. Improvement of Viewing Angle Dependence of Bottom-Emitting Green Organic Light-Emitting Diodes with a Strong Microcavity Effect. *Opt. Express* **2020**, *28* (21), 31686. https://doi.org/10.1364/oe.403398.

(14) Hall, D.; Suresh, S. M.; dos Santos, P. L.; Duda, E.; Bagnich, S.; Pershin, A.; Rajamalli, P.; Cordes, D. B.; Slawin, A. M. Z.; Beljonne, D.; Köhler, A.; Samuel, I. D. W.; Olivier, Y.; Zysman-Colman, E. Improving Processability and Efficiency of Resonant TADF Emitters: A Design Strategy. *Adv. Opt. Mater.* **2020**, *8*, 1901627. https://doi.org/10.1002/adom.201901627.

(15) Tenopala-Carmona, F.; Lee, O. S.; Crovini, E.; Neferu, A. M.; Murawski, C.; Olivier, Y.; Zysman-Colman, E.; Gather, M. C. Identification of the Key Parameters for Horizontal

Transition Dipole Orientation in Fluorescent and TADF Organic Light-Emitting Diodes. *Adv. Mater.* **2021**, *33* (37), 2100677. https://doi.org/10.1002/adma.202100677.

(16) Schmidt, T. D.; Lampe, T.; Daniel Sylvinson, M. R.; Djurovich, P. I.; Thompson, M. E.; Brütting, W. Emitter Orientation as a Key Parameter in Organic Light-Emitting Diodes. *Phys. Rev. Appl.* **2017**, *8* (3), 1–28. https://doi.org/10.1103/PhysRevApplied.8.037001.

(17) Campoy-Quiles, M.; Nelson, J.; Etchegoin, P. G.; Bradley, D. D. C.; Zhokhavets, V.; Gobsch, G.; Vaughan, H.; Monkman, A.; Ingänas, O.; Persson, N. K.; Arwin, H.; Garriga, M.; Alonso, M. I.; Herrmann, G.; Becker, M.; Scholdei, W.; Jahja, M.; Bubeck, C. On the Determination of Anisotropy in Polymer Thin Films: A Comparative Study of Optical Techniques. *Phys. Status Solidi Curr. Top. Solid State Phys.* **2008**, *5* (5), 1270–1273. https://doi.org/10.1002/pssc.200777835.
